# Gut microbiota-derived metabolites in cardiovascular disease: mechanisms, disease-specific roles, and translational opportunities

**DOI:** 10.3389/fcvm.2026.1884283

**Published:** 2026-07-23

**Authors:** Guanqun Zhu, Jianxiu Man

**Affiliations:** Department of Cardiology, Siping City Central People’s Hospital, Siping, China

**Keywords:** atherosclerosis, cardiovascular disease, gut microbiota-derived metabolites, short-chain fatty acids, translational medicine, trimethylamine n-oxide

## Abstract

Cardiovascular disease remains the leading cause of global morbidity and mortality and arises from complex interactions among metabolic dysregulation, inflammation, thrombosis, and vascular dysfunction. In recent years, the gut microbiota has emerged as an important regulator of cardiovascular pathophysiology, largely through the production of bioactive metabolites that act on distant organs. This review summarizes the major classes of gut microbiota-derived metabolites involved in cardiovascular disease, with particular emphasis on trimethylamine N-oxide, short-chain fatty acids, phenylacetylglutamine, bile acids, and tryptophan-derived metabolites. We discuss how these metabolites influence endothelial dysfunction, immune activation, lipid handling, platelet reactivity, cardiac remodeling, gut barrier integrity, and blood pressure regulation through interconnected signaling pathways. We further examine their disease-specific relevance in atherosclerosis, heart failure, hypertension, and coronary artery disease/acute coronary syndrome. In addition, we evaluate current translational strategies targeting microbial metabolism, including dietary modulation, probiotics and prebiotics, fecal microbiota transplantation, and selective inhibition of microbial enzymes. Rather than viewing individual metabolites as uniformly harmful or protective, we propose that cardiovascular risk is better understood as the net consequence of interacting microbial metabolic pathways within specific host contexts. This metabolite-centered framework may help refine biomarker development, risk stratification, and pathway-guided interventions in cardiovascular medicine.

## 1 Introduction

Cardiovascular diseases (CVDs) remain a leading cause of morbidity and mortality worldwide and arise through intertwined processes that include metabolic dysregulation, inflammation, thrombosis, and vascular dysfunction (1). Although traditional risk factors such as hypertension, dyslipidemia, diabetes mellitus, and smoking remain central to cardiovascular risk assessment, they do not fully explain disease heterogeneity or inter-individual differences in outcome, indicating that additional regulatory layers contribute to disease initiation and progression (2). According to the Global Burden of Disease (GBD) 2022 update, cardiovascular diseases remain the leading cause of death globally, accounting for approximately 19.8 million deaths in 2022, representing nearly one-third of all global mortality (3, 4). The burden of CVD continues to rise, particularly in low- and middle-income countries, driven by aging populations, increasing prevalence of metabolic risk factors, and persistent lifestyle-related exposures such as unhealthy diets, physical inactivity, obesity, and diabetes mellitus (5). In addition, recent epidemiological data indicate that the incidence of atherosclerotic cardiovascular disease and heart failure is increasing despite advances in pharmacological and interventional therapies, highlighting the urgent need for novel mechanistic insights and preventive strategies. Cardiovascular metabolomics has advanced rapidly with NMR and UPLC–MS technologies, enabling systematic profiling of metabolic alterations in cardiovascular disease (6). Recent studies in ischemic heart disease have reported metabolic disturbances, including elevated 3-hydroxybutyrate and succinate levels reflecting altered energy metabolism (7).

In this context, the gut microbiota has emerged as an important systemic regulator of host metabolism and immune homeostasis (8). Advances in metagenomics and metabolomics have shifted attention from microbial composition alone to microbial function, particularly the generation of bioactive metabolites that enter the circulation and influence distal organs. This functional perspective places microbial metabolites at the center of the gut-cardiovascular axis (9, 10).

Several classes of gut microbiota-derived metabolites are now linked to CVDs, including TMAO, SCFAs, PAGln, bile acids, and tryptophan-derived metabolites (11, 12). Rather than acting through a single pathway, these molecules converge on endothelial signaling, inflammatory networks, lipid metabolism, platelet reactivity, and tissue remodeling, thereby influencing the pathobiology of atherosclerosis, heart failure (HF), hypertension, and acute coronary syndrome (ACS). Importantly, the cardiovascular consequences of microbial metabolism are bidirectional: some metabolites amplify disease, whereas others preserve homeostasis.

Despite rapid progress, several questions remain unresolved. The context-dependent actions of individual metabolites across disease stages are incompletely defined, the interactions among parallel microbial metabolic pathways are not yet fully integrated, and many reported associations still lack rigorous causal validation. These gaps continue to limit clinical translation.

This review therefore synthesizes the major classes of gut microbiota-derived metabolites, the molecular mechanisms through which they influence cardiovascular biology, their disease-specific relevance, and the translational strategies currently being explored. By framing these data within a gut microbiota-metabolite-host response model, we aim to provide a more mechanistically integrated perspective on how microbial metabolism shapes cardiovascular disease and where the most realistic opportunities for precision intervention may lie.

**Figure 1:**
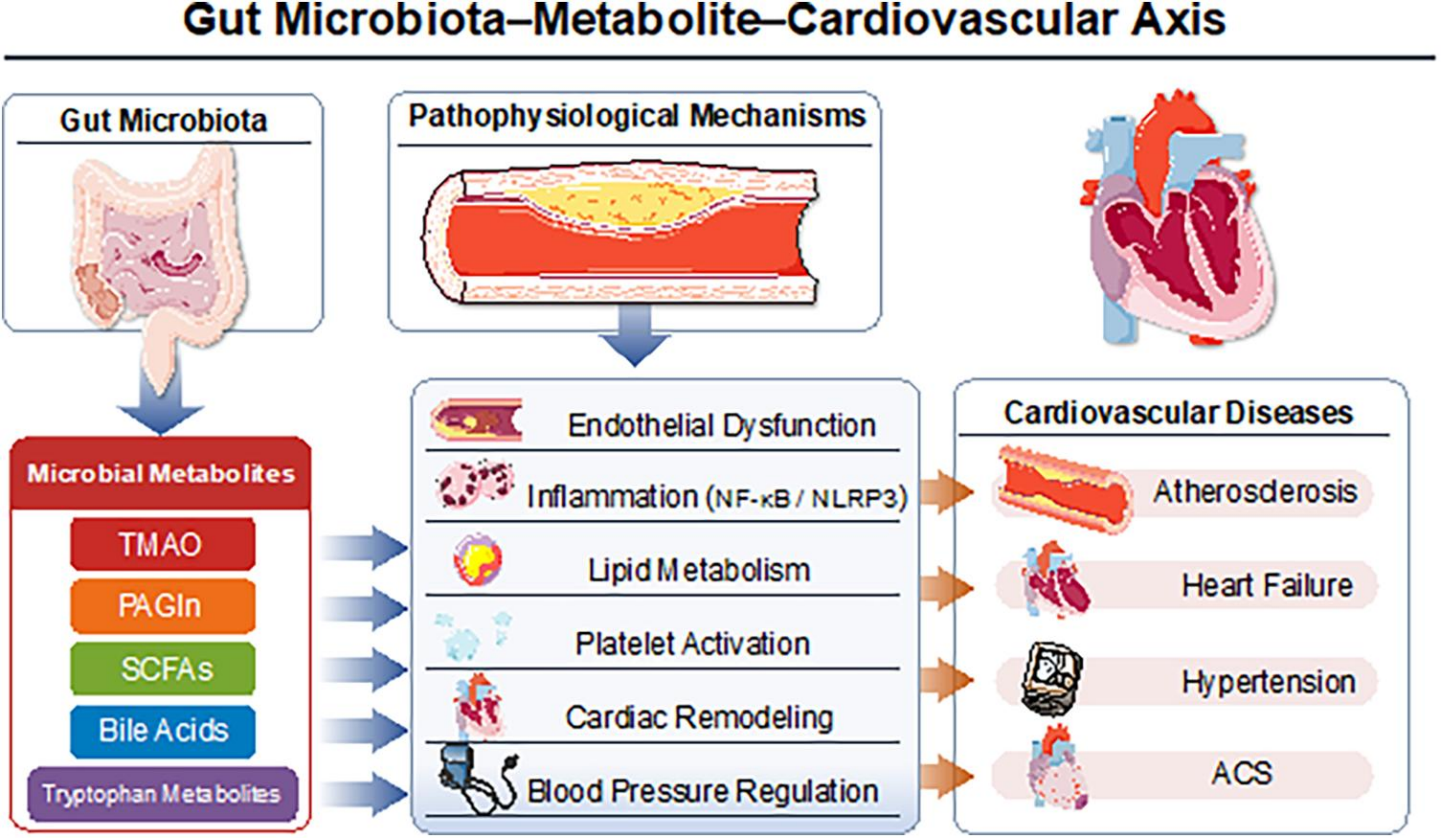
Overview of the gut microbiota-metabolite-cardiovascular axis.

To illustrate the integrated regulatory network linking gut microbiota-derived metabolites to cardiovascular pathophysiology, a schematic overview is presented in Figure 1.

The gut microbiota generates multiple classes of bioactive metabolites, including TMAO, PAGln, SCFAs, bile acids, and tryptophan-derived metabolites. After entering the circulation, these molecules influence endothelial dysfunction, inflammation, lipid handling, platelet activation, cardiac remodeling, and blood pressure regulation through interconnected signaling pathways such as NF-*κ*B and the NLRP3 inflammasome. Figure 1 summarizes this systems-level network and its relevance to major cardiovascular phenotypes.

## 2 Categories and functional characteristics of gut microbiota-derived metabolites

**Figure 2:**
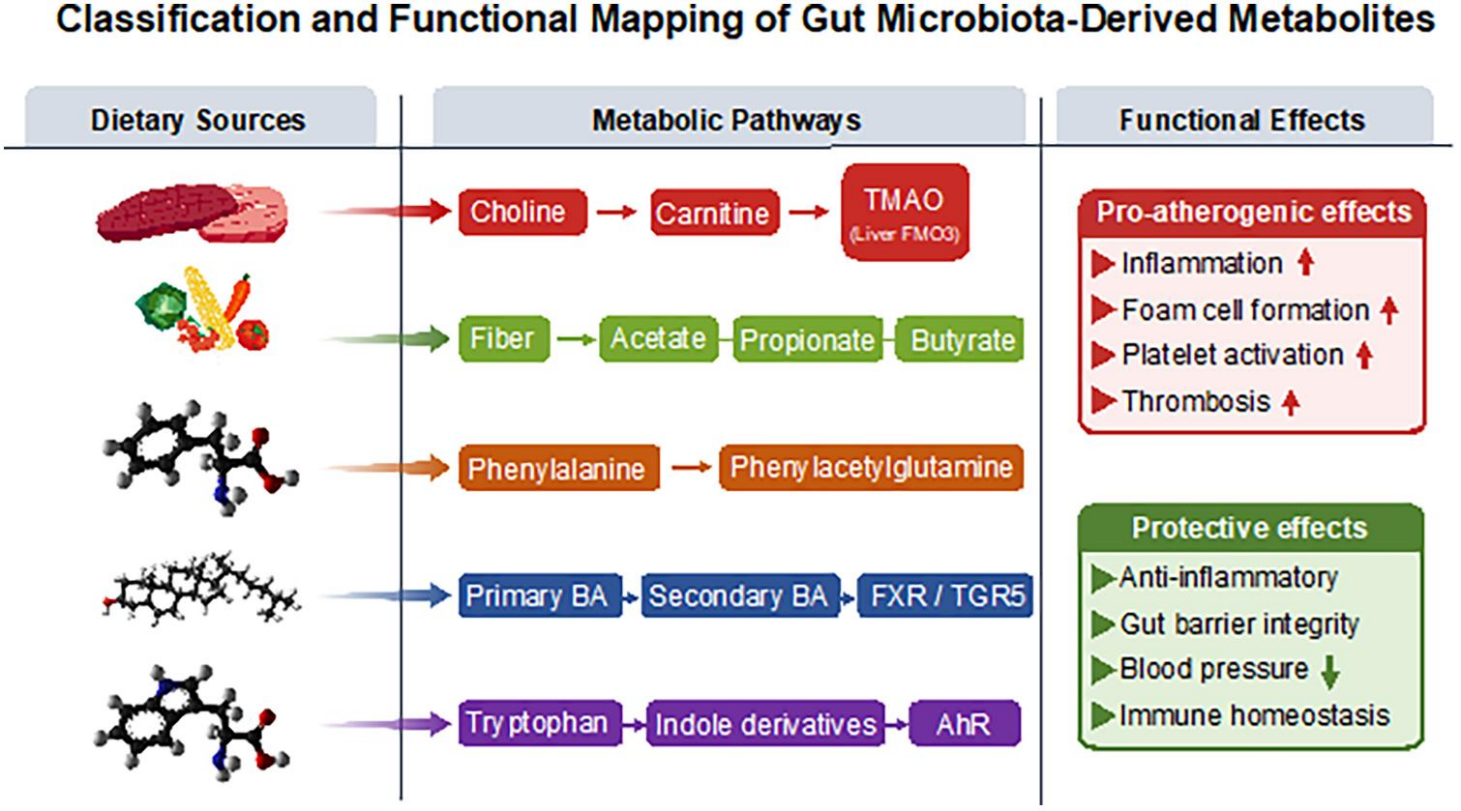
Classification and functional mapping of gut microbiota-derived metabolites.

Gut microbes convert dietary and host-derived substrates into several major metabolite classes with distinct but overlapping cardiovascular effects. TMAO and PAGln are most consistently linked to pro-atherogenic and pro-thrombotic signaling, whereas SCFAs, selected bile acids, and many tryptophan-derived indoles more often support barrier integrity, immune balance, and vascular homeostasis. Figure 2 maps these metabolites according to their origins, signaling routes, and predominant cardiovascular consequences.

Together, these metabolites form a functional interface between diet, microbial ecology, and host cardiovascular physiology. The following sections examine each major metabolite class in turn, with emphasis on mechanistic strength, context dependence, and translational relevance.

### 2.1 Trimethylamine N-oxide (TMAO)

TMAO is one of the most extensively studied gut microbiota-derived metabolites in cardiovascular research. Its generation depends on a serial metabolic process involving dietary substrates, the gut microbiota, and host hepatic enzymes. Nutrients containing trimethylamine moieties, such as choline, phosphatidylcholine, and L-carnitine, are first metabolized by the gut microbiota into trimethylamine (TMA). TMA then enters the liver via the portal vein and is further oxidized into TMAO by flavin-containing monooxygenase 3 (FMO3). Wang et al. first demonstrated systematically that dietary phosphatidylcholine significantly increases circulating TMAO levels after gut microbial metabolism, and this process was confirmed to be microbiota-dependent in germ-free or antibiotic-treated models (13). Koeth et al. subsequently showed that L-carnitine can likewise be metabolized by the gut microbiota into TMAO and can promote experimental atherosclerosis, indicating an important link between red meat-associated nutrients and the TMAO axis (14). Tang et al. further found in human studies that elevated circulating TMAO is independently associated with increased risk of major adverse cardiovascular events, thereby extending this metabolic pathway from mechanism to clinical risk stratification (15).

Mechanistically, the relationship between TMAO and atherosclerosis is first reflected in its regulation of cholesterol metabolism and foam cell formation. Early studies by Wang et al. showed that elevated TMAO has been shown to enhance macrophage cholesterol loading and foam cell formation in experimental models, suggesting its potential pro-atherogenic role (13). Subsequent studies suggested that TMAO enhances macrophage uptake of oxidized low-density lipoprotein (oxLDL) by upregulating scavenger receptor-related pathways and disrupts reverse cholesterol transport, thereby promoting lipid deposition and plaque progression. Geng et al. demonstrated in both *in vitro* and *in vivo* experiments that TMAO promotes foam cell formation via the CD36-dependent MAPK/JNK pathway, indicating that it is not merely an accompanying biomarker but may directly participate in plaque formation (16). In addition, TMAO can further amplify its pro-atherogenic effects by affecting bile acid metabolism and host cholesterol homeostasis.

Beyond lipid metabolic reprogramming, TMAO also exhibits marked pro-inflammatory and pro-thrombotic properties. Seldin et al. demonstrated that TMAO has been reported to activate MAPK and NF-*κ*B signaling pathways in vascular cells and smooth muscle cells, inducing inflammatory gene expression and leukocyte adhesion, thereby promoting the formation of a vascular inflammatory microenvironment (17). Chen et al. further showed that TMAO suppresses the SIRT3-SOD2 mitochondrial antioxidant axis, enhances mitochondrial reactive oxygen species (ROS) generation, and activates the NLRP3 inflammasome, ultimately aggravating vascular inflammation (18). Meanwhile, Zhu et al. reported in Cell that TMAO directly enhances platelet responsiveness to stimuli such as adenosine diphosphate (ADP), thrombin, and collagen, increases intracellular calcium release, and augments thrombus formation *in vivo* (19). Their cohort data also suggested that higher TMAO levels are associated with increased future thrombotic event risk. Together, these findings indicate that the role of TMAO in CVDs is not limited to chronic plaque formation, but rather spans the continuous pathological process of plaque progression, inflammatory amplification, and thrombotic triggering.

Despite strong mechanistic evidence supporting a pro-atherogenic and pro-thrombotic role of TMAO in experimental models, its clinical interpretation in human studies remains heterogeneous. Several large cohort studies have demonstrated that elevated circulating TMAO levels are associated with increased risk of major adverse cardiovascular events (20); however, other studies have reported that this association becomes attenuated or non-significant after adjustment for renal function, dietary intake, and metabolic comorbidities (21, 22). This inconsistency suggests substantial clinical heterogeneity in the predictive value of TMAO across different populations. In particular, circulating TMAO levels are strongly influenced by renal clearance capacity, habitual dietary patterns (especially intake of red meat and phosphatidylcholine-rich foods), gut microbial composition, and medication use (23). These factors may confound its interpretation as an independent cardiovascular risk biomarker. Therefore, TMAO should not be considered a universally stable predictive biomarker, but rather a context-dependent integrative signal reflecting host–microbiota metabolic interactions. This perspective helps reconcile the discrepancy between robust mechanistic evidence from preclinical studies and variable predictive performance observed in clinical cohorts.

Overall, although experimental studies support a pro-atherogenic role for TMAO, human evidence remains observational and inconsistent. Circulating TMAO is influenced by renal function, diet, metabolic status, and gut microbiota composition, suggesting that it may be better viewed as a context-dependent biomarker of host–microbiota metabolism than as an independent causal driver of cardiovascular disease.

### 2.2 Short-chain fatty acids (SCFAs)

SCFAs mainly include acetate, propionate, and butyrate, which are generated by gut microbial fermentation of dietary fiber, such as resistant starch, pectin, and non-starch polysaccharides. Unlike pathogenic metabolites such as TMAO, SCFAs are generally associated with beneficial cardiovascular effects in experimental and observational studies, and their actions mainly depend on both receptor-mediated signaling pathways and epigenetic regulatory mechanisms. The biological effects of SCFAs are also highly context-dependent, influenced by receptor distribution, disease state, dietary fiber intake, and host metabolic conditions such as obesity and diabetes.

At the receptor level, SCFAs participate in multiple physiological processes by activating G protein-coupled receptors (GPCRs), including GPR41 (FFAR3), GPR43 (FFAR2), and GPR109A. Maslowski et al. first demonstrated systematically that SCFAs regulate neutrophil chemotaxis and inflammatory responses via GPR43, and that the loss of this receptor leads to uncontrolled inflammation, underscoring the pivotal role of SCFAs in immune homeostasis (24). Further studies have shown that SCFAs can inhibit NF-*κ*B activation through GPR41/43 signaling, thereby reducing the expression of pro-inflammatory cytokines such as interleukin-6 (IL-6) and tumor necrosis factor-alpha (TNF-α), and alleviating chronic low-grade inflammation. This process is highly relevant to atherosclerosis and cardiovascular inflammation.

In blood pressure regulation, SCFAs are considered key signaling molecules linking the gut microbiota with host hemodynamics. Pluznick et al. found that SCFAs can lower blood pressure by acting on GPR41 in the vasculature and kidney, while at the same time exerting an opposing pressor effect through the olfactory receptor Olfr78, thus forming a finely tuned regulatory network (25). Marques et al. further confirmed in hypertensive animal models that a high-fiber diet or direct acetate supplementation significantly lowers blood pressure and improves cardiac remodeling, providing additional support for the cardiovascular protective role of SCFAs (26).

Regarding gut barrier function, butyrate is considered one of the most important energy substrates, serving as a major energy source for colonic epithelial cells and maintaining barrier integrity by promoting the expression of tight junction proteins such as occludin and claudin-1. Peng et al. showed that butyrate enhances intestinal epithelial barrier function by activating AMP-activated protein kinase (AMPK), thereby reducing lipopolysaccharide (LPS) translocation into the circulation and suppressing systemic inflammatory responses. This mechanism is particularly important for preventing cardiovascular injury driven by gut-derived inflammation (27).

Although SCFAs are widely considered protective metabolites in cardiovascular disease, their biological effects are highly context dependent. Most evidence supporting cardiovascular benefits derives from preclinical models, where controlled dosing and simplified microbial environments may not fully recapitulate human metabolic complexity (28). In human studies, associations between circulating or fecal SCFA levels and cardiovascular outcomes are less consistent. This inconsistency may be attributed to differences in dietary fiber intake, host receptor expression profiles (such as GPR41, GPR43, and Olfr78), gut microbial composition, and disease states such as obesity, diabetes, and chronic kidney disease (29). Moreover, SCFAs may exert divergent physiological effects depending on receptor distribution and tissue context, as illustrated by the opposing roles of vasodilatory and vasopressor signaling pathways. Therefore, SCFAs should be interpreted not as uniformly protective metabolites, but as pleiotropic signaling molecules whose net cardiovascular effects depend on host–microbiota–environment interactions.

At the epigenetic level, SCFAs, particularly butyrate and propionate, can act as histone deacetylase (HDAC) inhibitors and regulate gene expression. Chang et al. found that butyrate promotes regulatory T cell (Treg) differentiation by inhibiting HDAC activity, thereby enhancing immune tolerance and suppressing inflammation (30). In addition, SCFAs can regulate macrophage polarization through epigenetic mechanisms, shifting macrophages toward an anti-inflammatory phenotype and thereby exerting protective effects in atherosclerosis.

Notably, the physiological effects of SCFAs are concentration-dependent and tissue-specific, and they may display variable functions under different disease conditions and microbial backgrounds. For example, under metabolically dysregulated conditions, SCFA signaling pathways may be altered, thereby affecting the magnitude of their protective effects. Therefore, the role of SCFAs in CVDs should be understood as a dynamic regulatory network rather than a unidirectional protective factor.

Overall, SCFAs represent a counterbalancing arm of the gut microbial metabolic network. Through receptor-mediated signaling, epigenetic regulation, and preservation of barrier function, they help restrain inflammation and support vascular homeostasis (31). Their effects, however, remain context dependent and should be interpreted within the broader metabolic environment rather than as uniformly protective.

### 2.3 Phenylacetylglutamine (PAGln)

PAGln has recently emerged as a gut microbiota-derived metabolite of growing interest and is generated from the microbial metabolism of the aromatic amino acid phenylalanine. Specifically, the gut microbiota first converts phenylalanine into phenylacetic acid (PAA), which is then conjugated with glutamine in the host liver to generate PAGln and released into the circulation to exert biological effects. This host-microbiota co-metabolic pattern highlights the central role of the gut microbiota in regulating the host metabolic network. Moreover, PAGln-related platelet activation is likely modulated by host neurohumoral status, medication use (particularly beta-blockers), and metabolic disease states, further supporting its context-dependent biological effects. PAGly elevates calcium transients and sarcoplasmic reticulum calcium load, yet its action is weaker than adrenergic agonists. It also attenuates the cardiac response to sympathetic activation, linking this microbial metabolite to cardiovascular function (32).

Mechanistically, PAGln primarily influences platelet regulation and thrombosis propensity. Nemet et al. first showed that PAGln significantly enhances platelet responsiveness to multiple agonists, including ADP, thrombin, and collagen, and promotes arterial thrombosis in animal models (33). Further mechanistic work showed that PAGln activates beta-adrenergic receptors on platelets, particularly the beta2 receptor subtype, thereby triggering downstream cyclic adenosine monophosphate (cAMP)-dependent signaling and amplifying platelet activation. This finding directly links gut microbial metabolism with the classical neurohumoral regulatory system, suggesting that thrombosis is regulated not only by endogenous hormones but also by microbial metabolites.

Notably, the action of PAGln on platelets is characterized by a synergistic amplification effect. PAGln does not directly induce platelet aggregation by itself; rather, it markedly enhances platelet sensitivity to other physiological agonists, thereby amplifying thrombotic responses under conditions of vascular injury or inflammation. This characteristic is of particular significance in acute cardiovascular events such as myocardial infarction and ischemic stroke.

At the population level, elevated PAGln has been consistently associated with cardiovascular event risk. Nemet et al. found in a large cohort that higher circulating PAGln levels were independently associated with increased risk of major adverse cardiovascular events (MACE), even after adjustment for traditional risk factors. In addition, their study suggested that beta-blockers may partially reverse PAGln-induced platelet hyperreactivity, thereby providing a potential clue for clinical intervention.

From the perspective of mechanistic integration, PAGln and TMAO exhibit certain synergistic pro-thrombotic effects. Both enhance platelet activity, but through distinct pathways: TMAO mainly acts by augmenting calcium signaling and MAPK pathway activation, whereas PAGln acts through beta-adrenergic signaling. This multi-pathway cooperative amplification suggests that gut microbial metabolites may form a convergent signaling network in thrombosis, thereby markedly increasing the risk of acute cardiovascular events.

PAGln represents a recently identified gut microbiota-derived metabolite with emerging relevance to cardiovascular thrombosis. Although mechanistic studies have demonstrated its ability to enhance platelet reactivity through *β*-adrenergic receptor signaling, the current evidence base remains relatively limited compared with more established metabolites such as TMAO.

Importantly, most clinical data linking PAGln to cardiovascular outcomes are derived from observational cohorts, and interventional evidence is still lacking. It therefore remains unclear whether PAGln directly contributes to thrombotic disease pathogenesis or primarily reflects upstream alterations in amino acid metabolism, gut microbial activity, or host neurohumoral regulation.

In addition, potential interactions between PAGln and other pro-thrombotic pathways, as well as its modulation by medications such as beta-blockers, require further validation in larger, independent, and prospective studies. As such, PAGln should currently be considered a promising but early-stage biomarker rather than a fully established therapeutic target.

### 2.4 Bile acid metabolites

Bile acids (BAs) are not only essential molecules for lipid digestion, but also a class of hormone-like signaling molecules whose metabolism is highly dependent on gut microbial participation. Primary bile acids, such as cholic acid and chenodeoxycholic acid, are synthesized from cholesterol in the liver and secreted into the intestine, where they are converted by gut microbiota, mainly through 7*α*-dehydroxylation, into secondary bile acids such as deoxycholic acid and lithocholic acid, thereby forming a metabolic network coordinately regulated by the liver, intestine, and microbiota (34).

At the signaling level, bile acids mainly act through two classes of receptors: the nuclear receptor farnesoid X receptor (FXR) and the membrane receptor Takeda G protein-coupled receptor 5 (TGR5). FXR is widely expressed in the liver, intestine, and vascular tissues and is a core transcription factor regulating bile acid synthesis, lipid metabolism, and glucose homeostasis. Li et al. reported that FXR activation suppresses hepatic cholesterol synthesis and promotes reverse cholesterol transport, thereby exerting protective effects in atherosclerosis (35). In addition, FXR can reduce inflammatory responses by inhibiting NF-*κ*B signaling, further improving the vascular microenvironment.

Unlike FXR, TGR5 mainly mediates rapid non-genomic effects of bile acids. TGR5 activation promotes cAMP generation, thereby enhancing endothelial nitric oxide (NO) release and improving vasodilatory function. Activation of TGR5 in macrophages can also suppress inflammatory cytokine release and exert anti-inflammatory effects (36). Moreover, TGR5 participates in energy metabolism, including stimulation of brown adipose tissue thermogenesis and improvement of insulin sensitivity, thereby indirectly influencing cardiovascular risk.

Importantly, the gut microbiota indirectly regulates FXR/TGR5 signaling by modulating bile acid composition. Sayin et al. found that gut microbiota can alter the bile acid pool, particularly by regulating the proportion of FXR-antagonistic bile acids, thereby affecting host metabolic status. This process has major implications for lipid metabolism and glucose homeostasis (37). Jia et al. further proposed that bile acid-microbiota interactions exert bidirectional regulation in atherosclerosis, with different bile acid species potentially displaying either pro-inflammatory or anti-inflammatory effects (38).

In the context of CVDs, bile acid signaling is relatively complex. On the one hand, activation of FXR and TGR5 is generally considered beneficial through anti-inflammatory actions, improved lipid metabolism, and protection of vascular function. On the other hand, some secondary bile acids, such as lithocholic acid, may induce oxidative stress and cytotoxic responses at high concentrations, thereby aggravating tissue injury. Thus, the role of bile acids in CVDs is better understood as a dose- and composition-dependent bidirectional regulatory network.

Taken together, bile acids illustrate the complexity of host-microbiota co-metabolism in cardiovascular biology. Their effects depend not only on total abundance but also on bile acid composition, receptor context, and tissue exposure. This complexity makes the pathway biologically important but also argues for carefully stratified rather than one-size-fits-all therapeutic targeting.

### 2.5 Tryptophan-derived metabolites

Tryptophan (Trp) is an important metabolic substrate co-regulated by the gut microbiota and the host, and it is mainly metabolized through three pathways: the host-dominant kynurenine pathway, the serotonin pathway, and the gut microbiota-mediated indole pathway. Among these, microbiota-derived indole metabolites are regarded as important signaling molecules linking the gut microbiota with immune homeostasis and cardiovascular function (39).

The gut microbiota can metabolize tryptophan into multiple indole derivatives, including indole-3-acetic acid (IAA), indole-3-propionic acid (IPA), and indole-3-aldehyde (IAld). These molecules exert broad immunoregulatory effects through activation of the aryl hydrocarbon receptor (AhR). AhR is a ligand-dependent transcription factor expressed in intestinal epithelial cells and multiple immune cell types, and its activation is crucial for maintaining immune homeostasis.

With respect to immune regulation, indole metabolites promote Treg differentiation and suppress pro-inflammatory T helper 17 (Th17) responses through AhR signaling, thereby maintaining immune balance. Zelante et al. showed that microbiota-derived tryptophan metabolites promote interleukin-22 (IL-22) secretion through AhR, strengthening mucosal immune barriers and suppressing inflammation (40). Rothhammer et al. further confirmed that AhR signaling participates in inflammatory regulation in both the central nervous system and the peripheral immune system, highlighting the systemic immunomodulatory role of this pathway (41).

Indole molecules also play important roles in gut barrier function. Bansal et al. found that indole enhances the expression of intestinal epithelial tight junction proteins and suppresses inflammatory responses, thereby maintaining gut barrier integrity (42). This effect is important for preventing endotoxins such as LPS from entering the circulation and reducing systemic inflammation, which is a major driver of CVDs.

In the cardiovascular system, tryptophan metabolism exhibits complex bidirectional regulatory features. On the one hand, some indole derivatives, such as IPA, have antioxidant and anti-inflammatory properties and may exert protective effects by reducing oxidative stress and inflammation. On the other hand, the host-dominant kynurenine pathway is activated under inflammatory conditions, and its metabolites, such as kynurenine, are associated with endothelial dysfunction and increased cardiovascular risk. These observations suggest that branch selection within tryptophan metabolism may be reprogrammed under disease conditions, thereby influencing disease progression.

In addition, recent studies have shown that AhR signaling can directly affect endothelial function and metabolic homeostasis. Some AhR ligands can regulate oxidative stress responses and inflammatory signaling (43), thereby affecting vascular function and atherosclerotic progression. However, the role of this pathway varies considerably across ligands and tissues, and the specific mechanisms remain to be further investigated.

Overall, tryptophan-derived metabolites highlight how microbial metabolism can shape cardiovascular risk indirectly through immune regulation and barrier maintenance. Many indole derivatives appear protective, but their net effect depends on pathway balance, inflammatory state, and tissue context. This pathway therefore exemplifies both the promise and the complexity of targeting microbial metabolites in CVDs.

To provide a structured overview of the major gut microbiota-derived metabolites discussed in this review, their metabolic origins, signaling pathways, and cardiovascular effects are summarized in Table 1.

**Table 1:** Major gut microbiota-derived metabolites and gut-derived inflammatory mediators: metabolic origins, signaling pathways, and cardiovascular effects.

Metabolite	Major precursors/sources	Microbial and host metabolic pathway	Main receptors or signaling pathways	Major cardiovascular effects	Representative cardiovascular conditions
TMAO	Dietary choline, phosphatidylcholine, and L-carnitine; abundant in red meat, eggs, and some dairy products	Gut microbiota converts dietary trimethylamine-containing nutrients into trimethylamine (TMA), which is subsequently oxidized in the liver by flavin-containing monooxygenase 3 (FMO3) to generate TMAO	NF-*κ*B, NLRP3 inflammasome, MAPK/JNK, CD36-related lipid uptake pathways, calcium signaling in platelets	Promotes endothelial dysfunction, oxidative stress, vascular inflammation, foam cell formation, platelet hyperreactivity, thrombosis, and cardiac fibrosis/remodeling	Atherosclerosis, acute coronary syndrome, thrombosis-related events, heart failure
SCFAs	Dietary fiber, resistant starch, pectin, and non-starch polysaccharides	Gut microbial fermentation of indigestible carbohydrates produces acetate, propionate, and butyrate	GPR41 (FFAR3), GPR43 (FFAR2), GPR109A, AMPK, histone deacetylase (HDAC) inhibition; partial interaction with Olfr78 in blood pressure regulation	Exerts anti-inflammatory effects, enhances endothelial function, maintains gut barrier integrity, modulates immune homeostasis, lowers blood pressure, and attenuates cardiac remodeling	Hypertension, atherosclerosis, heart failure, metabolic cardiovascular disorders
PAGln	Phenylalanine-derived substrates	Gut microbiota metabolizes phenylalanine into phenylacetic acid (PAA), which is conjugated with glutamine in the host liver to form PAGln	*β*-adrenergic receptors, especially β2-adrenergic receptor-related signaling; downstream cAMP-dependent platelet activation pathways	Enhances platelet responsiveness to physiological agonists, amplifies thrombosis-related signaling, and increases susceptibility to acute cardiovascular events	Acute coronary syndrome, myocardial infarction, ischemic stroke, thrombotic cardiovascular disease
Bile acids	Cholesterol-derived primary bile acids synthesized in the liver	Primary bile acids are secreted into the intestine and transformed by gut microbiota into secondary bile acids through deconjugation and 7*α*-dehy droxylation reactions	Farnesoid X receptor (FXR), Takeda G protein-coupled receptor 5 (TGR5), NF-κB-related inflammatory signaling, metabolic regulatory pathways	Regulates lipid metabolism, cholesterol homeostasis, vascular inflammation, endothelial function, and systemic metabolic balance; effects may be protective or detrimental depending on bile acid composition and concentration	Atherosclerosis, metabolic cardiovascular disease, heart failure
Tryptophanderived metabolites	Dietary tryptophan	Tryptophan is metabolized by gut microbiota into indole derivatives such as indole-3-acetic acid (IAA), indole-3-propionic acid (IPA), and indole-3-aldehyde (IAld); host metabolism also contributes through kynurenine and serotonin pathways	Aryl hydrocarbon receptor (AhR), IL22-related mucosal immune signaling, redox and inflammatory regulatory pathways	Preserves gut barrier function, promotes immune tolerance, suppresses excessive inflammation, and may protect endothelial and vascular homeostasis; some pathway branches may also contribute to vascular dysfunction under inflammatory conditions	Atherosclerosis, hypertension, systemic inflammatory cardiovascular conditions
Gut-derived inflammatory mediators associated with barrier dysfunction (e.g., lipopolysaccharide, LPS)	Gram-negative bacterial cell Wall components; increased translocation under gut barrier dysfunction	Not a classical metabolite but a gut-derived inflammatory mediator that enters the circulation when intestinal permeability increases	TLR4, NF-κB, NLRP3 inflammasome	Triggers systemic inflammation, endothelial activation, vascular dysfunction, and inflammation-associated thrombosis	Atherosclerosis, heart failure, metabolic endotoxemia-associated cardiovascular disorders

TMAO, trimethylamine N-oxide; SCFAs, short-chain fatty acids; PAGln, phenylacetylglutamine; FXR, farnesoid X receptor; TGR5, Takeda G protein-coupled receptor 5; AhR, aryl hydrocarbon receptor; LPS, lipopolysaccharide.

Given the substantial heterogeneity in study designs and levels of evidence across gut microbiota-derived metabolites in cardiovascular disease, a structured framework is necessary to facilitate accurate interpretation of the current literature. While mechanistic insights are often derived from *in vitro* and animal studies, clinical relevance primarily depends on observational and interventional human evidence, which remains unevenly distributed across different metabolites. In particular, widely studied metabolites such as TMAO are supported by multiple lines of evidence, whereas emerging metabolites such as PAGln and certain tryptophan-derived products are still largely confined to preclinical or observational stages.

To address this limitation and improve clarity regarding the maturity of current evidence, we systematically categorized the major gut microbiota-derived metabolites according to the strength and type of supporting evidence, including *in vitro* studies, animal experiments, observational human studies, prospective cohort studies, and interventional clinical trials. The resulting framework is summarized in Table 2.

**Table 2:** Evidence strength of gut microbiota-derived metabolites in cardiovascular disease according to study type.

Metabolite	*In vitro* evidence	Animal studies	Observational human studies	Prospective cohort studies	Interventional clinical studies	Overall evidence level
TMAO	Strong (endothelial, macrophage, platelet models)	Strong (atherosclerosis, thrombosis models)	Strong (MACE, CAD, HF associations)	Moderate (risk prediction cohorts)	Limited (dietary modulation, indirect)	Moderate–high, but causality debated
SCFAs	Strong (epithelial, immune modulation)	Strong (BP, inflammation models)	Moderate (diet-associated levels)	Limited–moderate	Limited (fiber/probiotic trials)	Biologically strong, clinically heterogeneous
PAGln	Moderate (platelet activation pathways)	Moderate	Emerging (MACE association cohorts)	Limited	Very limited	Early-stage evidence
Bile acids	Strong (FXR/TGR5 signaling)	Moderate	Moderate	Limited	Limited	Context-dependent, mixed evidence
Tryptophan metabolites	Strong (AhR signaling, immune models)	Moderate	Emerging	Limited	Very limited	Mechanistically plausible, early translation

### 2.6 Evidence strength, causality, and unresolved controversies

Although gut microbiota-derived metabolites have been increasingly implicated in CVD, the strength and maturity of evidence vary substantially across metabolites and disease contexts. A key limitation in the current literature is the frequent conflation of mechanistic, observational, and interventional evidence, which may lead to overinterpretation of causality.

Among the major metabolites, TMAO is supported by relatively extensive mechanistic, animal, and human observational data, particularly in atherosclerosis, thrombosis, and heart failure. However, whether TMAO represents a causal mediator of cardiovascular pathology or primarily serves as a biomarker reflecting dietary intake, renal function, and gut microbial composition remains controversial. Confounding by renal clearance and interindividual variability in microbial TMA production further complicates causal inference (44).

SCFAs are strongly supported by experimental evidence demonstrating anti-inflammatory, barrier-protective, and blood pressure-regulating effects. Nevertheless, clinical translation remains heterogeneous, as their systemic effects are highly dependent on receptor distribution, host metabolic status, and dietary background. The bidirectional signaling via GPR41-mediated vasodilation and Olfr78-associated vasopressor effects further underscores their context-dependent nature (45, 46).

PAGln represents a recently identified metabolite with emerging evidence linking it to platelet hyperreactivity and thrombotic risk. While mechanistic studies suggest *β*-adrenergic receptor-mediated platelet activation, clinical evidence remains limited to observational cohorts, and its stability as a predictive biomarker requires further validation in prospective and interventional studies.

Bile acids and tryptophan-derived metabolites further illustrate the complexity of host–microbiota co-metabolism. Their cardiovascular effects are highly context dependent, influenced by receptor distribution, metabolic state, inflammatory milieu, and microbial composition, and may exert both protective and deleterious effects depending on physiological conditions (47).

Overall, current evidence supports a model in which gut microbiota-derived metabolites should not be interpreted as uniformly causal or protective entities. Instead, their cardiovascular effects are best understood within a dynamic framework that integrates metabolite interactions, host context, and disease stage. Stronger causal inference, standardized metabolomic profiling, and well-designed interventional studies are required before these metabolites can be translated into routine clinical tools.

A major limitation in current microbiome and metabolomics research is the lack of standardized protocols for metabolite quantification. Across existing studies, substantial methodological heterogeneity exists in sample collection (e.g., serum, plasma, feces), storage conditions, and processing workflows, which can significantly affect metabolite stability and measured concentrations (48). In addition, different analytical platforms, including LC–MS/MS, NMR-based metabolomics, and targeted vs untargeted approaches, often yield non-comparable results due to differences in sensitivity, specificity, and detection range. Furthermore, variability in data normalization, internal standards, and batch correction strategies further contributes to inter-study inconsistency (49). These methodological discrepancies limit the reproducibility and cross-cohort comparability of microbiota-derived metabolite measurements, thereby hindering the establishment of universal reference ranges and clinical thresholds. Therefore, the development of standardized, harmonized analytical pipelines is essential for improving the robustness and translational potential of microbiome-based metabolomic studies.

### 2.7 Context-dependent modulation of gut microbiota-derived metabolites

The cardiovascular effects of gut microbiota-derived metabolites are increasingly recognized to be highly context-dependent rather than uniform across individuals or disease states. This context dependency arises from the dynamic interplay between host factors and microbial metabolic activity.

Key host-related determinants include age-associated changes in microbiome diversity and metabolic capacity, sex-dependent differences in hormonal regulation of lipid and immune pathways, and dietary patterns that shape substrate availability for microbial metabolism (50, 51). In addition, disease states such as chronic kidney disease, diabetes mellitus, and obesity significantly alter systemic metabolite clearance, inflammatory tone, and gut microbial composition, thereby modifying circulating levels and biological activity of metabolites such as TMAO, SCFAs, and PAGln.

Furthermore, medication use (e.g., antibiotics, statins, beta-blockers, and metformin) can directly or indirectly reshape gut microbial communities and influence metabolite production (52, 53). Host genetic background also contributes to interindividual variability by regulating enzymatic pathways involved in microbial co-metabolism (e.g., FMO3-dependent TMAO synthesis) and receptor sensitivity to microbial metabolites (54).

Collectively, these factors determine not only the circulating levels of microbial metabolites but also tissue-specific receptor expression and downstream signaling responsiveness, ultimately shaping their net cardiovascular effects. Therefore, gut microbiota-derived metabolites should be interpreted within a host–microbiome–environment interaction framework rather than as fixed pathogenic or protective entities.

## 3 Key molecular mechanisms mediated by gut microbiota-derived metabolites

**Figure 3:**
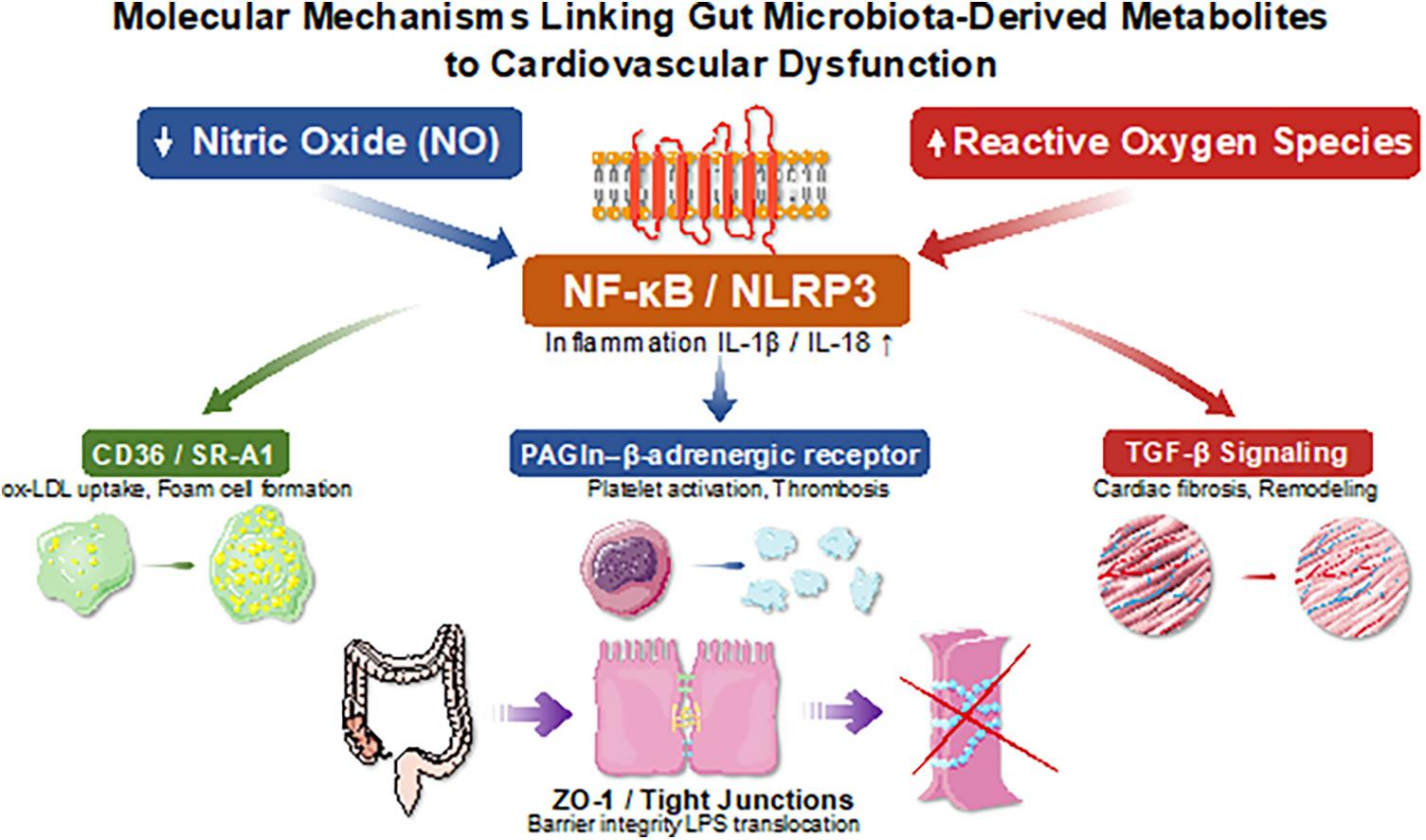
Molecular mechanisms linking gut microbiota-derived metabolites to cardiovascular dysfunction.

To illustrate the interconnected molecular network underlying gut microbiota-mediated cardiovascular regulation, a schematic overview is presented in Figure 3.

Gut microbiota-derived metabolites influence cardiovascular pathophysiology through an interconnected molecular network. Oxidative stress, impaired nitric oxide bioavailability, NF-*κ*B and NLRP3 activation, dysregulated lipid uptake, platelet hyperreactivity, fibrosis-related signaling, and barrier disruption are not isolated events; they reinforce one another across tissues. Figure 3 summarizes these convergent pathways and highlights how microbial metabolites can simultaneously shape vascular, immune, and cardiac phenotypes.

### 3.1 Endothelial dysfunction

Endothelial dysfunction is widely recognized as an early key event in the development and progression of atherosclerosis and multiple CVDs. Its core features include reduced NO bioavailability, enhanced oxidative stress, and activation of inflammatory responses. Recent studies indicate that gut microbiota-derived metabolites, particularly TMAO, play important regulatory roles in this process.

At the molecular level, TMAO can markedly reduce NO bioavailability by inducing oxidative stress. Sun et al. found that TMAO upregulates nicotinamide adenine dinucleotide phosphate oxidase expression in vascular endothelial cells, thereby promoting ROS generation and suppressing endothelial nitric oxide synthase (eNOS) activity, ultimately impairing vasodilatory function (55). In addition, TMAO can further enhance mitochondrial ROS production by inhibiting the SIRT3-SOD2 mitochondrial antioxidant pathway, thereby aggravating endothelial injury (18).

Besides oxidative stress, TMAO can also promote endothelial dysfunction through inflammatory signaling pathways. Seldin et al. showed that TMAO activates NF-*κ*B signaling and induces the expression of adhesion molecules such as vascular cell adhesion molecule-1 (VCAM-1) and intercellular adhesion molecule-1 (ICAM-1) in endothelial cells, thereby promoting monocyte adhesion and inflammatory cell infiltration (17). This process not only exacerbates local inflammation but also provides an important basis for atherosclerotic plaque formation.

Further studies have shown that TMAO activates the NLRP3 inflammasome and thereby promotes inflammatory cascades. Chen et al. demonstrated that TMAO activates the NLRP3 inflammasome by increasing mitochondrial ROS generation, thus promoting IL-1β release and aggravating vascular inflammation (18). This mechanism directly connects metabolic signals with innate immune responses and represents an important amplification loop in endothelial dysfunction.

By contrast, some gut microbiota-derived metabolites exert protective effects on endothelial function. For example, SCFAs can enhance eNOS activity through AMPK signaling and suppress oxidative stress, thereby improving vasodilatory function (26). In addition, SCFAs can indirectly protect endothelial cells by suppressing inflammatory pathways (56), highlighting the marked functional antagonism among different metabolites in endothelial regulation.

It should also be noted that endothelial dysfunction is not merely a local vascular event but is closely linked to systemic inflammation and metabolic disturbances. By regulating oxidative stress, inflammatory responses, and metabolic signaling, gut microbiota-derived metabolites establish an important functional connection between the intestine and the vasculature, making the endothelium a key target organ in the gut-cardiovascular axis.

Overall, gut microbiota-derived metabolites regulate endothelial function through the core mechanistic network of ROS generation, NO suppression, and inflammatory activation. The net outcome depends on the dynamic balance between pathogenic and protective metabolites. This framework not only explains the early occurrence of atherosclerosis but also provides a new theoretical basis for interventions targeting endothelial function.

### 3.2 Inflammation and immune modulation

Chronic low-grade inflammation is considered one of the core driving forces of CVD development and progression, and gut microbiota-derived metabolites play key roles in regulating immune homeostasis and inflammatory responses. These metabolites mediate both innate and adaptive immune responses through multiple signaling pathways, forming a metabolism-immune coupling network that influences vascular inflammation and cardiovascular pathology.

Among pro-inflammatory mechanisms, TMAO has been shown to activate inflammation through multiple signaling pathways. Seldin et al. demonstrated that TMAO activates NF-*κ*B signaling in vascular endothelial cells and smooth muscle cells, promoting the expression of inflammatory cytokines such as IL-6 and TNF-α, as well as adhesion molecules, thereby enhancing inflammatory cell recruitment (17). In addition, TMAO can activate the NLRP3 inflammasome by inducing oxidative stress, promoting maturation and release of IL-1β, and establishing a typical pro-inflammatory microenvironment (18). This process plays an important role in atherosclerotic plaque formation and progression.

In addition to TMAO, leakage of microbial products due to gut barrier dysfunction is another important mechanism amplifying inflammation. LPS, a typical endotoxin, can activate NF-*κ*B through Toll-like receptor 4 (TLR4), thereby inducing systemic inflammatory responses (57). This mechanism of gut-derived inflammation has been demonstrated in obesity, metabolic syndrome, and atherosclerosis, indicating that the gut microbiota participates in inflammatory networks not only through metabolites but also through barrier regulation.

By contrast, some gut microbiota-derived metabolites exhibit pronounced anti-inflammatory effects, among which SCFAs are the most representative. SCFAs can suppress NF-*κ*B signaling and reduce the release of pro-inflammatory cytokines through activation of GPR43 and GPR109A (58). In addition, SCFAs promote Treg differentiation by inhibiting HDAC activity, thereby maintaining immune tolerance and suppressing inflammation (59). This mechanism has been shown in atherosclerosis models to alleviate inflammation and plaque formation.

Tryptophan-derived metabolites, in turn, exert unique immunoregulatory effects through AhR signaling. Zelante et al. reported that microbiota-derived indole metabolites can act as AhR ligands and induce IL-22 production, thereby strengthening mucosal barrier function and suppressing inflammation (40). AhR signaling also participates in regulation of the Th17/Treg balance, a process that is crucial for immune homeostasis. Tryptophan-derived metabolites can regulate inflammatory responses in multiple organ systems, indicating broad systemic effects (41, 60).

Importantly, complex interactions exist among these metabolic pathways. For example, TMAO-induced inflammatory responses can further damage the gut barrier, thereby promoting LPS entry into the circulation and amplifying inflammation, whereas SCFAs and indole metabolites provide negative feedback by maintaining barrier integrity and suppressing inflammatory signaling. This dynamic balance between positive and negative regulation constitutes a central mechanism by which the gut microbiota regulates host immune homeostasis.

At the systemic level, gut microbiota-derived metabolites establish a continuous signaling network linking local, systemic, and vascular inflammation by regulating innate immunity, such as macrophage and dendritic cell activation, and adaptive immunity, such as T-cell differentiation. This cross-system immunoregulatory mechanism makes the gut microbiota an important distal regulator of inflammation in CVDs.

Overall, gut microbiota-derived metabolites form a dynamic balance between pro-inflammatory and anti-inflammatory signaling through key pathways including NF-*κ*B, NLRP3, and AhR. Disruption of this balance is an important mechanism driving chronic inflammation and CVD progression. This concept provides a new theoretical basis for modulating the immune microenvironment to intervene in CVDs.

### 3.3 Lipid metabolism and foam cell formation

Disturbed lipid metabolism and macrophage transformation into foam cells are central events in the development and progression of atherosclerosis. In this process, gut microbiota-derived metabolites influence cholesterol metabolism, lipid transport, and inflammatory responses. Among them, TMAO is considered a key molecule linking gut microbiota to host lipid metabolic reprogramming.

At the level of cholesterol transport, TMAO can significantly affect macrophage cholesterol homeostasis. Wang et al. first reported that TMAO promotes macrophage cholesterol accumulation and enhances foam cell formation (13). Subsequent studies further showed that TMAO suppresses the expression of ATP-binding cassette transporters ABCA1 and ATP-binding cassette subfamily G member 1 (ABCG1), thereby reducing cholesterol efflux, while upregulating scavenger receptors such as CD36 and SR-A1 to enhance oxLDL uptake and accelerate foam cell formation.

In addition to local macrophage effects, TMAO can also influence lipid metabolism systemically. Gut microbiota-mediated TMAO generation significantly suppresses reverse cholesterol transport and reduces cholesterol return from peripheral tissues to the liver (61, 62). Moreover, TMAO can disrupt cholesterol homeostasis further by modulating hepatic bile acid synthesis and related metabolic enzyme expression, thereby promoting atherosclerotic progression (16).

Inflammation and lipid metabolism are also closely interconnected. TMAO enhances inflammatory responses by activating NF-*κ*B signaling, and the resulting inflammatory environment can further promote lipid uptake and foam cell formation, thereby establishing an inflammation-lipid positive feedback loop (63). This mechanism helps explain why chronic low-grade inflammation significantly accelerates atherosclerotic progression.

By contrast, some gut microbiota-derived metabolites exert protective effects on lipid metabolism. For example, SCFAs can indirectly improve cholesterol homeostasis by regulating hepatic lipid metabolism and suppressing inflammation. Marques et al. showed that a high-fiber diet can reduce blood lipids and attenuate cardiovascular injury by increasing SCFA levels (26). In addition, bile acid signaling through FXR/TGR5 also plays important roles in regulating cholesterol metabolism and lipid homeostasis, and activation of these pathways promotes cholesterol clearance and suppresses lipid accumulation (36).

It should be emphasized that lipid metabolic regulation does not depend on a single metabolite, but rather reflects the combined effects of multiple gut microbial metabolites. TMAO, bile acids, and SCFAs can coordinately regulate cholesterol metabolism, inflammation, and energy balance through distinct pathways, thereby forming a complex regulatory network. Therefore, intervention at a single target may be insufficient to comprehensively correct lipid metabolic abnormalities.

At the systemic level, the gut microbiota acts as a distal metabolic regulator in atherosclerosis by controlling cholesterol metabolism, inflammatory responses, and energy homeostasis. Its central role lies in its influence on foam cell formation and plaque stability, thereby directly participating in disease progression.

Overall, gut microbiota-derived metabolites play key roles in the coupled network of lipid metabolism and inflammation by regulating cholesterol uptake, efflux, and reverse transport. This mechanism not only reveals the metabolic basis of atherosclerosis but also provides new directions for targeting microbial metabolic pathways to intervene in dyslipidemia.

### 3.4 Platelet activation and thrombosis

Platelet activation and thrombosis constitute the direct pathological basis of acute cardiovascular events such as myocardial infarction and ischemic stroke. Recent studies have shown that gut microbiota-derived metabolites play important roles in the regulation of platelet function and thrombus formation, making them a mechanistic bridge between the gut microbiota and acute cardiovascular events.

Among pro-thrombotic mechanisms, TMAO is considered the most representative metabolite. In a Cell study, Zhu et al. were the first to systematically demonstrate that TMAO markedly enhances platelet responsiveness to multiple agonists, including ADP, thrombin, and collagen, and promotes thrombosis *in vivo* (19). Mechanistically, TMAO amplifies platelet activation by enhancing intracellular Ca2 + signaling and activating the MAPK pathway. Their cohort data further showed a significant association between circulating TMAO levels and future thrombotic event risk, suggesting not only mechanistic importance but also potential clinical predictive value.

In addition to TMAO, PAGln represents another important microbial metabolite involved in platelet regulation through a different signaling route. Nemet et al. reported that PAGln enhances platelet responsiveness to physiological agonists by activating platelet beta2-adrenergic receptors, thereby significantly increasing thrombotic risk (33). Notably, PAGln acts primarily through signal amplification rather than direct induction of platelet aggregation, a property that gives it stronger pathogenic potential in the setting of inflammation or vascular injury.

From the perspective of mechanistic integration, TMAO and PAGln display clear synergistic effects in platelet regulation. TMAO mainly promotes platelet activation through enhancement of Ca2+-dependent signaling, whereas PAGln amplifies this process through beta-adrenergic signaling. This multi-pathway synergistic amplification suggests that gut microbial metabolites form a complex signaling network in thrombosis rather than acting through a single pathway.

Inflammation also plays an important role in thrombosis. By activating NF-*κ*B and NLRP3 inflammatory pathways, gut microbiota-derived metabolites promote the release of inflammatory cytokines, thereby enhancing platelet activity and vascular endothelial adhesion (17, 18). This inflammation-thrombosis coupling mechanism is particularly important in atherosclerotic plaque rupture and thrombus formation.

It is worth noting that some microbial metabolites may indirectly protect against thrombosis. For example, SCFAs can reduce platelet activation tendency by suppressing inflammation and improving endothelial function, thereby inhibiting thrombosis to some extent (64). However, this effect is relatively indirect and still requires further mechanistic clarification.

From a systemic perspective, the gut microbiota regulates platelet function through metabolites, indicating that thrombus formation does not depend solely on local vascular factors, but is also influenced by whole-body metabolic status. This insight expands the pathogenesis of acute cardiovascular events from a local vascular injury model to a systemic metabolism-inflammation regulation model.

Overall, gut microbiota-derived metabolites play key roles in thrombosis by regulating platelet activation and inflammatory responses. TMAO and PAGln, through distinct signaling pathways, cooperatively enhance platelet reactivity and thus provide an important mechanistic basis linking the gut microbiota with acute cardiovascular events. This mechanism offers potential targets for the development of new antithrombotic strategies.

### 3.5 Gut barrier dysfunction and systemic inflammation

The gut barrier is an important structure that maintains homeostasis between the host and intestinal microorganisms. Its integrity is essential for limiting the entry of microbes and microbial products into the circulation. Recent studies have shown that dysbiosis and altered microbial metabolites can impair gut barrier function and induce gut-derived inflammation, a process that plays a key role in the development and progression of CVDs.

Structurally, the gut barrier is mainly composed of intestinal epithelial cells and their tight junctions, including proteins such as occludin, claudin, and ZO-1. This barrier not only provides physical separation but also maintains host homeostasis through immunoregulation. When dysbiosis or inflammation occurs, tight junction protein expression decreases, leading to increased intestinal permeability, namely the leaky gut state (65).

Functionally, impaired gut barrier integrity allows bacterial products to enter the circulation, among which LPS is one of the most important inflammatory triggers. Cani et al. introduced the concept of metabolic endotoxemia, indicating that mild but persistent increases in LPS can induce chronic low-grade inflammation through activation of the TLR4/NF-*κ*B pathway, thereby promoting metabolic disorders and CVDs (66). This concept first connected gut barrier dysfunction with systemic inflammation and metabolic disease.

In the context of CVDs, gut barrier dysfunction is particularly evident. For example, in HF patients, intestinal hypoperfusion and venous congestion can aggravate epithelial hypoxia and barrier disruption, thereby promoting translocation of bacteria and bacterial products into the circulation and establishing a vicious cycle linking the gut, inflammation, and HF (67). Similar mechanisms have also been observed in atherosclerosis and metabolic syndrome, indicating that gut barrier dysfunction is one of the common pathological foundations of multiple cardiovascular disorders.

Gut microbiota-derived metabolites exert bidirectional effects on gut barrier function. On the one hand, SCFAs, especially butyrate, can promote tight junction protein expression through AMPK activation and provide energy for intestinal epithelial cells, thereby maintaining barrier integrity (68). SCFAs can also indirectly protect the barrier by suppressing inflammation. On the other hand, pro-inflammatory metabolites and inflammatory signals, such as TMAO-induced inflammation, may aggravate gut barrier damage and form positive feedback amplification.

In addition, tryptophan-derived metabolites play important roles in barrier maintenance through AhR signaling. Zelante et al. showed that microbiota-derived indole molecules promote IL-22 production via AhR, thereby strengthening epithelial barrier function and suppressing inflammatory responses (40). This mechanism further illustrates that microbial metabolites regulate the interaction between immune function and barrier integrity.

At the systemic level, gut barrier dysfunction not only allows inflammatory factors to enter the circulation, but also establishes a continuous pathogenic pathway linking the gut, immune system, and vasculature by activating immune responses, promoting vascular inflammation, and enhancing platelet reactivity. In this way, the gut becomes an important signaling source influencing distal organs such as the cardiovascular system.

Overall, the gut barrier functions as a gatekeeper in CVDs. Its disruption can lead to gut-derived inflammation and metabolic dysregulation, thereby accelerating disease progression. Gut microbiota-derived metabolites play central roles in this process by regulating barrier integrity and inflammatory responses. This mechanism not only explains the distal connection between intestinal biology and CVDs, but also provides new directions for barrier-targeted interventions.

### 3.6 Cardiac remodeling and fibrosis

Cardiac remodeling is a key pathological process by which multiple CVDs progress to HF. It is characterized mainly by myocardial hypertrophy, interstitial fibrosis, inflammation, and cardiomyocyte apoptosis. Recent evidence indicates that gut microbiota-derived metabolites play important roles in cardiac remodeling by regulating inflammation, oxidative stress, and fibroblast activation.

Among pathogenic mechanisms, TMAO is considered an important metabolic factor promoting cardiac remodeling. Li et al. found that TMAO promotes cardiac fibroblast activation and collagen deposition by activating the TGF-*β*/Smad3 signaling pathway, thereby accelerating cardiac fibrosis (69). In addition, TMAO can exacerbate myocardial structural remodeling by inducing oxidative stress and inflammation and promoting cardiomyocyte hypertrophy and apoptosis.

*In vivo*, Organ et al. demonstrated in mouse models that elevated TMAO significantly aggravates pressure overload-induced myocardial hypertrophy and fibrosis and promotes deterioration of cardiac function (70). Their study further suggested that inhibition of TMAO production, for example by targeting gut microbial metabolic pathways, can significantly improve cardiac structure and function, indicating the importance of microbial metabolism in HF.

Beyond direct effects, the gut microbiota can indirectly promote cardiac remodeling through gut barrier dysfunction and inflammation. As noted above, gut-derived LPS entering the circulation can activate TLR4/NF-*κ*B signaling, thereby inducing myocardial inflammation and promoting fibrosis. In patients with HF, this mechanism is particularly pronounced because of intestinal hypoperfusion and barrier disruption, forming a vicious gut-inflammation-HF cycle.

By contrast, some gut microbiota-derived metabolites protect against cardiac remodeling. SCFAs can alleviate myocardial injury through anti-inflammatory and antioxidant effects. For example, acetate and butyrate may inhibit inflammation and improve myocardial energy metabolism through GPR43 and AMPK signaling, thereby attenuating myocardial hypertrophy and fibrosis (71). In addition, SCFAs can suppress inflammation-driven cardiac remodeling by modulating immune cell function.

Bile acid signaling also participates in cardiac remodeling. Activation of FXR and TGR5 may exert indirect cardioprotective effects by regulating metabolic homeostasis and inflammatory responses. However, certain bile acids may induce oxidative stress and cytotoxicity at high concentrations and thereby aggravate myocardial injury, suggesting again that their roles are bidirectional (72).

Importantly, the gut microbiota shows clear systemic regulatory characteristics in HF. On the one hand, microbial metabolites can act directly on cardiomyocytes and fibroblasts; on the other hand, they indirectly influence cardiac remodeling by regulating inflammation, gut barrier function, and metabolic homeostasis. This multi-level regulation makes the gut microbiota an important distal regulator in HF progression.

Overall, gut microbiota-derived metabolites play key roles in cardiac remodeling by regulating inflammation, oxidative stress, and fibrotic signaling pathways. Among them, TMAO promotes fibrosis through pathways such as TGF-*β*/Smad3, whereas metabolites such as SCFAs exert protective effects. This dynamic pathogenic-protective balance provides an important theoretical basis for understanding HF pathogenesis and for developing new intervention strategies.

### 3.7 Blood pressure regulation

Hypertension is one of the most common cardiovascular risk factors, and its development involves multiple mechanisms, including neurohumoral regulation, vascular function, and metabolic status. In recent years, gut microbiota-derived metabolites, especially SCFAs, have been shown to play important roles in blood pressure regulation, making the gut-blood pressure axis a new research hotspot.

At the molecular level, SCFAs are thought to regulate blood pressure in part through activation of GPCRs, among which GPR41 and GPR43 are the most important. Pluznick et al. found that SCFAs lower blood pressure by activating GPR41, possibly through modulation of sympathetic activity and vasodilation (25). In addition, SCFAs can act on endothelial cells to enhance NO generation and thus improve vasodilation. Notably, SCFAs also exert bidirectional effects on blood pressure regulation. In addition to their GPR41-mediated hypotensive effects, they activate the olfactory receptor 78 (Olfr78), which is expressed in the kidney and vascular smooth muscle and can elevate blood pressure by promoting renin release. This antagonistic “GPR41-mediated depressor vs. Olfr78-mediated pressor” mechanism suggests that SCFAs form a finely balanced dynamic regulatory system in blood pressure control.

At the systemic level, the gut microbiota can also regulate blood pressure through the renin-angiotensin system (RAS). Yang et al. found that dysbiosis can lead to RAS activation and vascular dysfunction, thereby promoting hypertension (73). In addition, SCFAs can indirectly lower blood pressure by suppressing inflammation and oxidative stress and thus improving vascular function.

Dietary factors are also important in this process. A high-fiber diet promotes SCFA production and improves blood pressure and cardiovascular function. Marques et al. reported that dietary fiber and acetate supplementation significantly lower blood pressure and improve cardiac structure, highlighting the key role of gut microbial metabolism in dietary intervention (26). This finding provides an important basis for improving cardiovascular health by modulating dietary patterns.

Gut microbial metabolites may also regulate blood pressure by influencing the immune system. For example, inflammatory responses can promote hypertension by damaging the endothelium and enhancing vasoconstrictive reactivity, whereas SCFAs may counteract this process by suppressing inflammation.

Overall, blood pressure regulation by the gut microbiota involves neuroregulation, vascular function, the renin system, and immune responses. This multi-pathway cooperation makes the gut microbiota an important distal regulator of blood pressure.

In summary, SCFAs play central roles in blood pressure regulation through receptors such as GPR41, GPR43, and Olfr78 and form a multilayer regulatory network by modulating inflammatory responses and vascular function. Changes in SCFA levels caused by dysbiosis may disrupt this balance and thereby promote hypertension. This mechanism provides a new theoretical basis for the development of gut microbiota-based antihypertensive strategies.

## 4 Roles in different cardiovascular diseases

### 4.1 Atherosclerosis

Atherosclerosis is the common pathological basis of most CVDs, and its development involves multiple mechanisms, including endothelial dysfunction, lipid deposition, inflammation, and thrombosis. Recent studies indicate that gut microbiota-derived metabolites regulate these key processes through multiple pathways and thus play important roles in atherosclerosis, with TMAO being the most representative pathogenic metabolite.

At the level of lipid metabolism, TMAO promotes plaque formation by regulating cholesterol transport. In the landmark Nature study by Wang et al., gut microbiota-mediated TMAO production was shown to significantly promote atherosclerotic plaque formation (13). The mechanisms include suppression of reverse cholesterol transport and modulation of macrophage cholesterol metabolism. Further studies found that TMAO promotes foam cell formation by upregulating scavenger receptors such as CD36 and SR-A1 while inhibiting ABCA1/ABCG1-mediated cholesterol efflux, thereby accelerating lipid deposition and plaque progression (14).

In terms of inflammation, TMAO induces inflammatory cytokine release and promotes immune cell recruitment by activating NF-*κ*B and NLRP3 inflammasome pathways, thereby amplifying the local inflammatory microenvironment (17, 74). This inflammatory response not only promotes plaque formation but also affects plaque stability, rendering plaques more prone to rupture. Moreover, LPS translocation caused by gut barrier dysfunction can further amplify inflammation, establishing a positive feedback loop between gut-derived and vascular inflammation.

At the endothelial level, TMAO reduces NO bioavailability by inducing oxidative stress and suppressing eNOS activity, which impairs vasodilatory function (74). As an early event in atherosclerosis, endothelial dysfunction creates favorable conditions for lipid deposition and inflammatory cell adhesion.

In addition, platelet activation is critical in plaque rupture and acute thrombus formation. Zhu et al. showed that TMAO markedly enhances platelet reactivity and promotes thrombosis (19), suggesting that it contributes not only to plaque formation but also directly to acute event risk.

Besides TMAO, other microbial metabolites also participate in atherosclerotic regulation. PAGln has been shown to enhances platelet activity through *β*-adrenergic receptors and increases thrombotic risk (33), whereas SCFAs and certain bile acids exert protective effects against atherosclerosis through anti-inflammatory actions, improved lipid metabolism, and maintenance of endothelial function (75).

Overall, the gut microbiota establishes a multilayer regulatory network in atherosclerosis through its metabolites, including the following pathways:

Lipid metabolic reprogramming -> foam cell formation
Inflammatory activation -> plaque progression
Endothelial dysfunction -> early vascular injury
Platelet activation -> acute thrombotic events

This multi-pathway cooperative model indicates that the gut microbiota not only participates in atherosclerosis onset but also influences its progression and clinical outcomes.

In summary, atherosclerosis represents the clearest example of how microbial metabolites can integrate dyslipidemia, endothelial injury, inflammation, and thrombosis into a single disease process. TMAO remains the most consistently implicated mediator, but the overall phenotype likely reflects the balance between harmful and compensatory microbial pathways. This framework supports microbiota-informed risk stratification while also underscoring the need for mechanistic and interventional validation.

### 4.2 Heart failure

HF is the terminal stage of multiple cardiovascular disorders, and its progression involves cardiac remodeling, inflammation, metabolic dysregulation, and abnormal neurohumoral regulation. In recent years, the role of the gut microbiota in HF has drawn increasing attention, and the gut-cardiovascular axis has been recognized as an important mechanistic pathway linking the intestinal microenvironment with cardiac functional changes.

At the metabolic level, TMAO is considered a key metabolite closely associated with HF. Tang et al. showed that elevated circulating TMAO levels are significantly associated with increased mortality risk and adverse prognosis in patients with HF (76). Further studies indicated that TMAO is not only predictive but also directly involved in HF progression. A separate meta-analysis likewise showed that elevated plasma TMAO levels in HF patients are associated with poor prognosis (77). Organ et al. demonstrated in animal experiments that TMAO aggravates pressure overload-induced myocardial hypertrophy and fibrosis and worsens cardiac function (70). The underlying mechanisms mainly involve activation of the TGF-*β*/Smad3 signaling pathway, enhancement of inflammatory responses, and promotion of oxidative stress.

With respect to inflammation and immunity, patients with HF commonly exhibit chronic low-grade inflammation. Gut microbiota-derived metabolites, especially TMAO and LPS, can promote inflammatory responses by activating NF-*κ*B and NLRP3 inflammasome pathways, thereby exacerbating myocardial injury (78). In addition, inflammation can promote cardiomyocyte apoptosis and fibroblast activation, further driving cardiac remodeling.

Gut barrier dysfunction is another important mechanism in HF. Sandek et al. found that because of intestinal hypoperfusion and venous congestion, patients with HF often develop intestinal mucosal hypoxia and barrier disruption, leading to entry of bacteria and bacterial products into the circulation (79). This process induces gut-derived inflammation, further aggravates systemic inflammation and cardiac dysfunction, and forms a typical vicious cycle linking the gut, inflammation, and HF.

In terms of metabolism and energy regulation, the gut microbiota influences host energy metabolism via metabolites and thereby indirectly affects cardiac function. For example, SCFAs may exert cardioprotective effects by improving metabolic homeostasis and suppressing inflammation (80). SCFAs may also reduce inflammation-driven myocardial injury by modulating immune responses.

Importantly, the role of the gut microbiota in HF displays clear bidirectional regulatory characteristics. On the one hand, pathogenic metabolites such as TMAO aggravate HF by promoting inflammation and fibrosis; on the other hand, protective metabolites such as SCFAs and certain bile acids exert beneficial effects through anti-inflammatory and metabolic regulatory actions. This dynamic balance determines the overall effect of the gut microbiota in HF.

From a systems perspective, HF is not merely a local cardiac disease but rather a systemic syndrome. By regulating inflammation, metabolic status, and gut barrier function, the gut microbiota establishes a complex interaction network among the intestine, immune system, and heart. This insight extends the pathological model of HF from a traditional heart-centered model to a multi-organ interaction model.

Overall, HF should be viewed not only as a cardiac disorder but also as a systemic syndrome shaped by gut barrier dysfunction, inflammatory signaling, and microbial co-metabolism. TMAO is the most consistently associated pathogenic metabolite, whereas SCFAs and selected bile acid pathways may be protective. Future progress will depend on separating correlation from causation and identifying the patient subsets most likely to benefit from microbiota-directed intervention.

### 4.3 Hypertension

Hypertension is one of the most prevalent cardiovascular risk factors worldwide, and its development involves multiple mechanisms, including vascular dysfunction, neurohumoral dysregulation, and immune-inflammatory responses. Recent studies have shown that gut dysbiosis and altered microbial metabolites play important roles in the development of hypertension, making the gut-blood pressure axis an emerging research focus.

Both human and animal studies have linked alterations in gut microbial composition to hypertension. Hypertensive patients and animal models exhibit marked dysbiosis characterized by reduced microbial diversity and depletion of beneficial bacteria (81). Moreover, transplantation of gut microbiota from hypertensive animals into healthy recipients can induce blood pressure elevation in the recipients, further confirming the pathogenic role of the gut microbiota in hypertension (82).

At the molecular level, SCFAs are core metabolites involved in blood pressure regulation. SCFAs participate in blood pressure control through activation of GPR41 and GPR43. Activation of GPR41 is associated with hypotensive effects, whereas Olfr78 promotes renin release and thereby elevates blood pressure (25). This bidirectional hypotensive-hypertensive regulatory mechanism suggests that SCFAs maintain a finely tuned dynamic balance in blood pressure regulation.

In addition to direct receptor signaling, SCFAs can lower blood pressure by improving endothelial function and suppressing inflammation. SCFAs enhance NO generation and suppress oxidative stress, thereby improving vasodilatory function. At the same time, they attenuate vascular injury by suppressing inflammatory responses through inhibition of NF-*κ*B signaling (83). Furthermore, SCFAs can indirectly influence blood pressure by regulating immune cells, such as Tregs, and maintaining immune homeostasis.

The gut microbiota can also participate in blood pressure regulation by modulating the RAS. Studies have shown that dysbiosis promotes RAS activation, thereby leading to vasoconstriction and elevated blood pressure (84). In addition, inflammation and oxidative stress can further enhance vascular responsiveness to angiotensin II and exacerbate hypertensive progression.

Dietary factors also play important roles. A high-salt diet not only directly raises blood pressure but can also worsen hypertension by altering gut microbiota composition, for example by decreasing Lactobacillus abundance, and by promoting inflammatory responses (85). Conversely, a high-fiber diet can significantly lower blood pressure and improve cardiovascular function by increasing SCFA production (86).

Furthermore, gut barrier dysfunction and gut-derived inflammation also participate in hypertension. Increased intestinal permeability can lead to LPS entry into the circulation and activation of the TLR4/NF-*κ*B pathway, thereby inducing inflammation and promoting blood pressure elevation. This mechanism further illustrates that the gut microbiota regulates blood pressure through multiple layers of interconnected pathways.

Overall, the gut microbiota regulates blood pressure through metabolites and involves neuroregulation, vascular function, immune inflammation, and metabolic homeostasis. The core of this process lies in SCFA-mediated receptor signaling and inflammatory regulation, whereas metabolite alterations caused by dysbiosis may disrupt this balance and promote hypertension.

In summary, hypertension illustrates how microbial metabolites can influence cardiovascular physiology beyond plaque biology by modulating vascular tone, immune activation, and neurohumoral signaling. SCFAs appear central to this axis, but their net effect depends on receptor context, host diet, and microbial composition. These features make microbiota-based antihypertensive strategies promising, but they also highlight the need for individualized rather than uniform approaches.

### 4.4 Coronary artery disease and acute coronary syndrome (CAD/ACS)

Coronary artery disease (CAD) and its acute manifestation, acute coronary syndrome (ACS), represent the direct clinical consequences of atherosclerotic progression and plaque rupture. In recent years, gut microbiota-derived metabolites have been demonstrated to play important roles in the occurrence, progression, and prognosis of CAD/ACS, making them a crucial bridge linking metabolic abnormalities with acute cardiovascular events.

At the epidemiological level, elevated TMAO levels are closely associated with the risk of CAD and ACS. Tang et al. found that plasma TMAO levels are significantly associated with the severity of coronary artery disease and future MACE risk (15). Further large cohort studies confirmed that TMAO is not only a risk marker but also an independent predictor, with elevated levels significantly increasing the risks of myocardial infarction, stroke, and death (13).

Mechanistically, gut microbiota-derived metabolites participate in CAD/ACS development through multiple pathways. First, during plaque formation, TMAO promotes foam cell formation and lipid deposition by regulating lipid metabolism (14). Second, during plaque progression, TMAO amplifies local inflammation and affects plaque stability by activating NF-*κ*B and NLRP3 inflammatory pathways (18). Together, these effects promote the formation of vulnerable plaques.

With respect to acute event triggering, platelet activation and thrombosis are the direct causes of ACS. Zhu et al. demonstrated that TMAO markedly enhances platelet reactivity and promotes thrombus formation, thereby increasing the risk of acute events (19). In addition, PAGln enhances platelet function through *β*-adrenergic receptor activation and cooperates in thrombosis (33). This metabolite-platelet axis provides a new explanation for ACS occurrence.

The gut microbiota can also contribute to ACS through gut barrier dysfunction and systemic inflammation. Barrier disruption allows LPS to enter the circulation and activate the TLR4/NF-*κ*B pathway, thereby enhancing inflammation and promoting plaque instability. Inflammatory responses can also increase platelet activity and endothelial injury, thereby further promoting thrombosis.

Importantly, some gut microbiota-derived metabolites exert protective effects in CAD/ACS. For example, SCFAs can attenuate atherosclerotic progression and reduce acute event risk through anti-inflammatory actions, improved endothelial function, and immunomodulation (87). Bile acids may also exert protective effects in CAD by regulating lipid metabolism and inflammation through the FXR/TGR5 signaling pathway (88, 89).

Overall, the gut microbiota establishes a multi-stage regulatory model in CAD/ACS:

Plaque formation: disturbed lipid metabolism (TMAO)
Plaque progression: enhanced inflammation (NF-*κ*B/NLRP3)
Plaque rupture: endothelial dysfunction and inflammation
Acute events: platelet activation and thrombosis (TMAO/PAGln)

This continuous process indicates that the gut microbiota not only participates in chronic disease progression but also directly influences the occurrence of acute events.

In summary, CAD and ACS reflect the point at which chronic metabolic and inflammatory disturbances translate into acute thrombotic events. Within this continuum, TMAO and PAGln appear particularly relevant because they link plaque biology to platelet hyperreactivity and clinical instability. Their translational value may ultimately lie in combined use for risk stratification and pathway-guided intervention rather than as standalone markers.

## 5 Translational opportunities and challenges in targeting gut microbiota-derived metabolites

Although a wide range of microbiota-directed strategies has shown cardiovascular potential, their translational maturity varies substantially. Dietary modulation and selected probiotic interventions have already entered human studies, whereas fecal microbiota transplantation and targeted inhibition of microbial metabolic enzymes remain largely experimental. In addition, emerging approaches based on host enzyme modulation, circulating metabolite profiling, and microbiome-guided patient stratification may provide more precise interventions but also introduce challenges related to causality, safety, standardization, and regulatory approval. This section therefore evaluates not only the therapeutic rationale of these approaches but also their current clinical readiness and major barriers to implementation.

Table 3 summarizes the disease-specific roles, mechanistic features, and translational implications of the major gut microbiota-derived metabolites across representative cardiovascular conditions.

**Table 3:** Roles of gut microbiota-derived metabolites in different cardiovascular diseases and their potential therapeutic implications.

Cardiovascular disease	Key microbial metabolites involved	Major mechanisms	Pathophysiological consequences	Potential therapeutic strategies
Atherosclerosis	TMAO, SCFAs, bile acids, tryptophan-derived metabolites	TMAO promotes foam cell formation, endothelial dysfunction, oxidative stress, and vascular inflammation; SCFAs suppress inflammation and improve barrier integrity; bile acids regulate lipid metabolism through FXR/TGR5; tryptophan metabolites modulate immune balance through AhR	Plaque initiation and progression, lipid deposition, chronic vascular inflammation, endothelial injury, and plaque instability	High-fiber dietary intervention, modulation of TMA/TMAO production, probiotics/prebiotics, microbiota-directed metabolic intervention, bile acid signaling modulation
Heart failure	TMAO, SCFAs, bile acids, gut-derived inflammatory mediators including LPS	TMAO promotes cardiac remodeling and fibrosis through inflammatory and profibrotic signaling such as TGF-β/Smad3; gut barrier dysfunction increases systemic inflammation; SCFAs may attenuate inflammation and improve metabolic homeostasis	Myocardial hypertrophy, fibrosis, ventricular remodeling, worsening cardiac dysfunction, and progression to heart failure	Dietary regulation, microbiota-targeted reduction of TMAO production, reinforcement of gut barrier integrity, anti-inflammatory interventions, precision metabolite-guided risk management
Hypertension	SCFAs, tryptophan-derived metabolites, bile acids	SCFAs regulate blood pressure through GPR41, GPR43, and Olfr78, influence vascular tone and renin signaling, and reduce inflammation; tryptophan metabolites may affect vascular and immune homeostasis; bile acids may indirectly influence metabolic and vascular regulation	Increased vascular resistance, endothelial dysfunction, immune-mediated vascular injury, and blood pressuredysregulation	High-fiber diets, SCFA-promoting nutritional strategies, probiotics/prebiotics, microbiota-guided antihypertensive interventions
ACS	TMAO, PAGln, gut-derived inflammatory mediators	TMAO enhances platelet calcium signaling and thrombogenicity; PAGln amplifies platelet reactivity via β-adrenergic receptor signaling; inflammatory mediators intensify endothelial activation and thrombo-inflammatory responses	Plaque rupture-related thrombosis, acute ischemic events, amplified platelet activation, and higher risk of recurrent cardiovascular events	Antithrombotic strategies informed by microbial metabolite profiling, inhibition of TMA/TMAO-related pathways, modulation of PAGln-associated signaling, dietary and microbiota-based risk reduction
Thrombosis-related cardiovascular events	TMAO, PAGln	TMAO enhances platelet responsiveness and thrombosis propensity; PAGln increases platelet sensitivity to physiological agonists through adrenergic signaling; both may act synergistically in thrombo-inflammatory settings	Arterial thrombosis, heightened thrombotic risk, recurrent ischemic events	Small-molecule inhibition of microbial trimethylamine production, beta-adrenergic pathway modulation, dietary intervention, personalized risk stratification based on metabolite profiles
Metabolic cardiovascular disorders	SCFAs, bile acids, TMAO, tryptophan-derived metabolites	SCFAs regulate immune and metabolic homeostasis; bile acids coordinate lipid and glucose metabolism; TMAO links dietary nutrient metabolism to vascular injury; tryptophan metabolites affect inflammation and barrier function	Chronic low-grade inflammation, dyslipidemia, endothelial dysfunction, and increased cardiovascular susceptibility	Diet-based microbiota remodeling, prebiotics/probiotics, fecal microbiota transplantation in selected contexts, integrated metabolomics-guided intervention
Systemic inflammation-associated cardiovascular injury	LPS-associated gut-derived inflammatory signals, TMAO, tryptophan-derived metabolites, SCFAs	Barrier dysfunction permits translocation of inflammatory microbial products; TMAO amplifies inflammatory signaling; SCFAs and indole derivatives counteract inflammation by preserving barrier integrity and immune tolerance	Persistent systemic inflammation, vascular injury, dysfunction, and acceleration of cardiovascular disease progression	Restoration of gut barrier function, anti-inflammatory microbiota modulation, SCFA-enhancing nutritional strategies, targeted microbiome intervention

### 5.1 Dietary interventions

Diet is the most accessible and scalable lever for modulating gut microbial metabolism. By altering substrate availability, dietary patterns can shift the relative production of harmful and protective metabolites and thereby influence cardiovascular risk. For this reason, dietary intervention remains the most mature entry point for targeting the gut microbiota-metabolite-cardiovascular axis.

To reduce pathogenic metabolites, restricting the intake of foods rich in choline and L-carnitine, such as red meat and egg yolk, can decrease TMAO production. Studies have shown that long-term red meat consumption significantly increases TMAO levels through gut microbial metabolism and thereby promotes atherosclerosis (90). In addition, phosphatidylcholine in the diet is likewise an important precursor of TMAO, and its metabolism depends on the gut microbiota (15). Reducing excessive intake of TMA-containing precursors may lower TMAO exposure in selected individuals; however, whether this reduction independently translates into fewer cardiovascular events remains uncertain.

To increase protective metabolites, a high-fiber diet is one of the most important interventions. Dietary fiber can be fermented by gut microbiota into SCFAs, which exert multiple actions including anti-inflammatory effects, improvement of endothelial function, and regulation of blood pressure. Marques et al. reported that a high-fiber diet and acetate supplementation significantly lower blood pressure and improve cardiac structure (26). In addition, SCFAs can improve the cardiovascular microenvironment by promoting Treg differentiation and suppressing inflammatory responses (91).

Dietary patterns play a central role in shaping the gut microbiota–derived metabolite profile and determining the overall metabolic balance. In Western dietary patterns, characterized by high intake of red meat, processed foods, and choline- and carnitine-rich nutrients, there is a shift toward increased production of pro-atherogenic metabolites such as TMAO and PAGln, which collectively promote inflammatory activation, endothelial dysfunction, and pro-thrombotic states. In contrast, high-fiber dietary patterns are associated with increased production of SCFAs, including acetate, propionate, and butyrate. These metabolites enhance gut barrier integrity, suppress inflammatory signaling, and improve endothelial function, thereby shifting the metabolic environment toward a protective phenotype. In real-world populations, such dietary transitions have been consistently associated with reduced cardiometabolic risk, supporting the clinical relevance of diet-induced modulation of microbial metabolite profiles.

At the dietary pattern level, the Mediterranean diet is considered one of the most representative cardioprotective dietary models. This diet is rich in fiber, unsaturated fatty acids, and polyphenols, and can significantly improve gut microbiota composition and reduce inflammation. De Filippis et al. found that long-term adherence to the Mediterranean diet increases the abundance of SCFA-producing bacteria, such as Faecalibacterium prausnitzii, thereby improving metabolic status (92). Meanwhile, polyphenols can also be metabolized by the gut microbiota into active products with antioxidant and anti-inflammatory properties, further contributing to cardiovascular protection.

In addition, a high-salt diet adversely affects both gut microbiota and cardiovascular health. As noted above, high salt intake can reduce Lactobacillus abundance and aggravate hypertension by promoting Th17 responses. This finding suggests that dietary intervention influences not only metabolite generation but also cardiovascular disease through immune regulation.

At the same time, dietary effects are highly individualized. Baseline microbiota composition, habitual diet, medication exposure, and host metabolic status all influence how a given person converts food-derived substrates into circulating metabolites. Future dietary strategies will therefore need to move beyond generic advice toward microbiome-informed precision nutrition.

Overall, diet regulates the gut microbiota and its metabolites at multiple levels, including metabolism, inflammation, and vascular function. Its central mechanisms include the following:

Reducing pathogenic metabolite production (e.g., TMAO)
Increasing protective metabolite levels (e.g., SCFAs)
Modulating immune responses and gut barrier function

Overall, dietary intervention remains the most practical near-term strategy for reshaping gut microbial metabolism in CVDs. Its strengths are safety, scalability, and compatibility with existing cardiovascular prevention programs; its limitation is variable individual responsiveness. Integration of dietary counseling with microbiome and metabolomic profiling may therefore be especially valuable.

### 5.2 Probiotics, prebiotics, and synbiotics

Probiotics, prebiotics, and synbiotics provide a more directed way to modulate microbial composition and function than diet alone. In principle, these strategies can suppress harmful metabolite generation, enhance SCFA production, improve barrier integrity, and temper inflammation, making them attractive adjuncts in cardiovascular prevention and management.

Mechanistically, probiotics act through multiple pathways. First, probiotics can competitively inhibit pathogenic bacterial colonization and thereby reduce the generation of pro-inflammatory metabolites, including precursors of TMAO. Probiotic Bifidobacterium has been reported to reduce serum TMAO levels in patients with unstable angina through the gut-liver-heart axis (93). In addition, some probiotics, such as Lactobacillus and Bifidobacterium species, can improve lipid metabolism and reduce cardiovascular risk by regulating bile acid metabolism and cholesterol absorption (94, 95).

Second, probiotics can exert anti-inflammatory and metabolic regulatory effects by promoting SCFA production. Prebiotics, such as inulin and fructooligosaccharides, serve as fermentable substrates for gut microbiota and can significantly increase SCFA production, thereby improving blood pressure, suppressing inflammation, and strengthening gut barrier function (96, 97). This process establishes an important functional connection among the microbiota, metabolites, and host.

In terms of immune regulation, probiotics can suppress inflammation by modulating immune cell function. For example, certain probiotics can promote Treg differentiation and inhibit Th17 responses, thereby reducing chronic inflammation (98). In addition, probiotics can improve gut barrier function by enhancing tight junction protein expression and thereby reduce systemic inflammation by limiting LPS translocation (99).

Clinical studies have reported modest improvements in selected cardiovascular risk markers following probiotic interventions, although effects on hard cardiovascular outcomes remain unproven. Randomized controlled trials have demonstrated that probiotic supplementation can significantly reduce serum total cholesterol and low-density lipoprotein cholesterol (LDL-C) levels (100). However, most trials have been relatively small, have used heterogeneous strains and doses, and have focused on surrogate endpoints rather than major cardiovascular events. Some studies have also reported mild reductions in blood pressure and improvements in inflammatory markers (101). Although these effects are relatively modest, their safety and long-term application potential make them important adjunctive strategies.

Synbiotics, defined as combined application of probiotics and prebiotics, can further enhance intervention efficacy through synergistic actions. Prebiotics provide substrates for probiotics, thereby improving probiotic colonization and metabolic activity and enhancing SCFA production and anti-inflammatory effects. This combination strategy may achieve higher efficiency and stability in microbiota modulation.

However, enthusiasm for probiotic-based strategies should be tempered by several unresolved issues. Effects are strain specific rather than class wide, colonization durability is variable, and clinical responses are influenced by the pre-existing microbiota and host environment. Accordingly, future studies should prioritize mechanistic strain selection, dose optimization, and patient stratification rather than treating probiotics as interchangeable agents.

Overall, probiotics, prebiotics, and synbiotics act at multiple levels, including metabolism, inflammation, and barrier integrity, by regulating gut microbiota structure and microbial metabolites. Their core mechanisms include:

Suppressing pathogenic metabolite generation (e.g., TMAO)
Promoting production of protective metabolites such as SCFAs
Improving gut barrier function and immune homeostasis

Taken together, probiotics, prebiotics, and synbiotics remain promising but heterogeneous tools. Their greatest translational potential likely lies in targeted, phenotype-specific applications supported by mechanistic biomarkers rather than in broad empirical supplementation.

### 5.3 Fecal microbiota transplantation and targeted metabolic inhibition

Compared with dietary modulation or conventional probiotic supplementation, fecal microbiota transplantation (FMT) and targeted inhibition of microbial metabolic pathways intervene more directly at the level of microbial ecosystem structure or function. These approaches therefore offer greater mechanistic precision but also raise greater challenges related to safety, reproducibility, and implementation. Studies demonstrate that the gut microbiome governs host metabolic health via microbial metabolites, gut barrier regulation and multi-organ axis signaling, and its dysbiosis contributes to obesity, type 2 diabetes and other metabolic disorders (102). Conventional microbiome and probiotic interventions face efficacy limitations due to host-microbe-environment heterogeneity, while multi-omics and machine learning technologies support the development of precision, personalized probiotic therapy and microbiome-targeted metabolic interventions (103).

#### 5.3.1 Fecal microbiota transplantation

FMT restores intestinal microbial homeostasis by transferring donor microbiota to a recipient. It is already clinically established for recurrent Clostridioides difficile infection (104), and its broader appeal lies in the possibility of replacing a metabolically harmful microbial ecosystem with one that generates a more favorable metabolite profile. In cardiovascular disease, however, this concept remains exploratory rather than established.

Mechanistically, FMT can reduce pathogenic metabolite generation and enhance protective metabolite production by restoring microbial diversity and function. Studies have found that transplantation of fecal microbiota from healthy donors into patients with metabolic syndrome significantly improves insulin sensitivity and alters gut microbiota composition (105, 106). In addition, animal studies have shown that transplantation of healthy microbiota into atherosclerosis models can suppress atherosclerotic progression (107).

In hypertension, Li et al. found that transplantation of microbiota from hypertensive animals into normotensive animals induces blood pressure elevation, whereas transplantation of healthy microbiota improves blood pressure (108). These results suggest that FMT may have therapeutic potential in CVDs through regulation of microbial structure and metabolic function.

Despite its conceptual appeal, FMT faces substantial translational barriers in CVDs, including donor selection, product standardization, durability of engraftment, and infection risk. At present, it is best regarded as an experimental strategy that may help establish causality, rather than as a near-term routine cardiovascular therapy.

#### 5.3.2 Microbial enzyme-targeted inhibition

Targeted inhibition of microbial metabolism offers a more reductionist and potentially more controllable approach. Instead of broadly remodeling the ecosystem, this strategy seeks to block the generation of specific pathogenic metabolites at their enzymatic source.

Among these approaches, inhibition of the TMA/TMAO pathway has been studied most extensively. Wang et al. developed choline analog compounds, such as 3,3-dimethyl-1-butanol (DMB), which inhibit microbial TMA lyase activity, thereby reducing TMA and TMAO generation and significantly attenuating atherosclerosis (109). This study was the first to demonstrate that targeting microbial metabolic enzymes can effectively intervene in cardiovascular disease progression.

New choline TMA lyase inhibitors, including iodomethylcholine (IMC) and fluoromethylcholine (FMC), have subsequently been developed (110). Further studies have shown that these inhibitors are non-lethal, meaning that they do not markedly disrupt the overall microbial structure but selectively inhibit specific metabolic functions, thereby reducing the risk of side effects. This function-targeted strategy offers clear advantages over conventional antibiotics.

In addition, recent studies have attempted to intervene in disease by regulating other metabolic pathways, such as bile acid metabolism and tryptophan metabolism. For example, modulation of FXR/TGR5 signaling or the AhR pathway may influence lipid metabolism and inflammatory responses and thereby improve cardiovascular function. However, these strategies remain at an early stage of investigation.

Antibiotics can reduce TMAO generation by suppressing the gut microbiota and have therefore been useful as proof-of-concept tools in mechanistic studies (78). However, their lack of specificity, microbiota-disrupting effects, and potential to promote antimicrobial resistance make them unsuitable as long-term cardiovascular therapies.

Therefore, antibiotics are more suitable as research tools than as long-term therapeutic strategies, which further highlights the advantages of targeted inhibition of microbial metabolic pathways.

The translational appeal of microbial TMA lyase inhibition lies in its ability to suppress a disease-associated microbial function without broadly eliminating the gut microbiota. However, the available evidence for DMB, IMC, and FMC remains predominantly preclinical. Their long-term effects on microbial ecology, compensatory metabolic pathways, hepatic TMAO production, and cardiovascular outcomes in humans have not yet been adequately established. Pharmacokinetic optimization, dose selection, potential off-target effects, and regulatory classification of microbiome-directed small molecules also require further evaluation. Therefore, microbial TMA lyase inhibitors represent one of the most mechanistically advanced microbiota-targeted strategies, but they cannot yet be considered clinically validated cardiovascular therapies.

### 5.4 Host-targeted modulation of the TMAO pathway

In addition to suppressing microbial TMA production, modulation of the host enzyme FMO3 has been proposed as an alternative strategy for reducing systemic TMAO exposure. Because FMO3 catalyzes the hepatic conversion of TMA into TMAO, genetic silencing or pharmacological inhibition of FMO3 could theoretically reduce circulating TMAO independently of the composition of the gut microbiota (111).

However, FMO3 is involved in a broader network of hepatic metabolic processes, including xenobiotic oxidation, lipid homeostasis, glucose metabolism, and bile acid regulation. Consequently, direct FMO3 inhibition may produce systemic metabolic effects that extend beyond the TMA/TMAO pathway. Excessive suppression may also lead to TMA accumulation and trimethylaminuria-like adverse effects. These concerns substantially narrow the therapeutic window and distinguish host-directed FMO3 inhibition from microbial TMA lyase inhibition (112).

At present, FMO3-targeted strategies remain primarily supported by experimental studies, and evidence from cardiovascular clinical trials is lacking. Future development will require tissue-selective or pathway-selective approaches, careful metabolic safety assessment, and demonstration that lowering TMAO through FMO3 modulation improves clinical outcomes rather than merely altering a circulating biomarker.

### 5.5 Metabolomics-guided risk stratification and microbiome-based precision medicine

Circulating microbial metabolites may provide complementary information beyond conventional cardiovascular risk factors. TMAO and PAGln have been associated with adverse cardiovascular events, whereas SCFA and bile acid profiles may reflect barrier integrity, dietary patterns, and host metabolic status. Integrating these metabolites into multi-analyte panels may therefore improve cardiovascular phenotyping and help identify patient subgroups characterized by enhanced thrombotic, inflammatory, or metabolic risk (113).

However, the clinical value of metabolomics-guided risk stratification remains uncertain. Most available studies have evaluated individual metabolites in observational cohorts, and there is no universally accepted concentration threshold, analytical platform, or reference range for routine cardiovascular use (114). Metabolite levels are also affected by renal function, recent dietary exposure, medication use, sampling conditions, and microbial composition. Consequently, the incremental predictive value of microbial metabolites over established risk models must be demonstrated through external validation, calibration analyses, and assessment of clinical utility.

Microbiome-based precision medicine extends this concept from risk prediction to intervention selection. Baseline microbial composition may influence whether an individual responds to dietary fiber, probiotics, prebiotics, or precursor-restriction strategies. Combining metagenomic, metabolomic, dietary, and clinical data could therefore support personalized selection of interventions. Nevertheless, such approaches currently remain exploratory because microbiome signatures are population dependent, longitudinal stability is uncertain, and reproducible treatment-response algorithms have not yet been established.

### 5.6 Current clinical evidence and barriers to implementation

Human studies targeting the gut microbiota–metabolite axis remain limited in scale and design. Dietary and probiotic interventions represent the most clinically advanced approaches, but most trials have focused on surrogate outcomes such as circulating TMAO, lipid levels, blood pressure, inflammatory markers, or microbial composition rather than major cardiovascular events. For example, a recent small study in patients with unstable angina reported reductions in circulating TMAO following one month of Bifidobacterium supplementation; however, the intervention cohort included only 10 patients, lacked a parallel randomized control group, and did not assess cardiovascular outcomes (93). A small randomized trial of Lactobacillus plantarum in patients with atherosclerotic cardiovascular disease has also examined changes in circulating TMAO, but such preliminary studies remain insufficient to establish clinical efficacy (115).

Observational clinical studies have further evaluated TMAO as a prognostic marker in myocardial infarction and coronary artery disease, but these studies do not constitute direct testing of microbiome-targeted therapy. Importantly, microbial TMA lyase inhibitors and FMO3-targeted agents have not yet generated robust cardiovascular outcome data in humans. Thus, the current clinical landscape is characterized by early proof-of-concept studies and biomarker-focused trials rather than definitive evidence of reduced cardiovascular events. Recent clinical studies have also highlighted the translational potential of microbial metabolite-based biomarkers, although their clinical implementation remains limited by variability in measurement standardization and population heterogeneity (116, 117).

Several barriers must be addressed before microbiota-derived metabolites can be incorporated into routine cardiovascular practice. First, causal pathways remain incompletely established, and lowering a biomarker does not necessarily translate into clinical benefit. Second, microbiome and metabolomic measurements lack harmonized protocols for sampling, storage, sequencing, quantification, and data normalization. Third, microbial metabolism varies substantially according to diet, renal function, age, sex, medication use, comorbidities, and geographic background. Fourth, long-term safety, durability of microbiome modification, potential compensatory pathways, and interactions with standard cardiovascular therapies remain insufficiently characterized. Finally, regulatory pathways for live biotherapeutics, fecal products, microbiome-directed small molecules, and multi-omics diagnostic tests remain heterogeneous. Large, multicenter, prospectively designed trials with standardized endpoints will therefore be required before these approaches can be incorporated into cardiovascular guidelines.

A major challenge in current microbiome–cardiovascular research is the difficulty in distinguishing causal relationships from simple associations. Although numerous studies have reported significant correlations between gut microbiota-derived metabolites and cardiovascular disease phenotypes, most of the available evidence is derived from observational or cross-sectional studies, which inherently limits causal inference. These associations are often influenced by multiple confounding factors, including dietary habits, host genetic background, medication use, renal function, and environmental exposures. As a result, it remains difficult to determine whether observed metabolic alterations are direct drivers of cardiovascular pathology or secondary consequences of disease progression. Furthermore, while preclinical studies provide important mechanistic insights into microbiota-derived metabolites, the translation of these findings into clinical practice remains challenging. Inter-individual variability in gut microbiota composition, metabolic capacity, and host response significantly limits the generalizability of experimental results. Therefore, careful interpretation is required when extrapolating mechanistic findings to human populations. Overall, these limitations highlight the need for well-designed longitudinal and interventional studies to better establish causality and improve the translational value of microbiome-based cardiovascular research.

## 6 Future directions and knowledge gaps in gut microbiota-derived cardiovascular research

Despite substantial progress in elucidating the role of gut microbiota-derived metabolites in CVD, several fundamental limitations continue to constrain translation from mechanistic insight to clinical application.

First, a major limitation lies in the predominance of associative evidence. Most human studies are cross-sectional or observational in nature, making it difficult to determine whether gut microbiota-derived metabolites are causal drivers of cardiovascular pathology or merely reflective biomarkers of diet, renal function, and host metabolic status. Although experimental models provide mechanistic support, interventional studies in humans remain limited, and definitive causal inference is still lacking.
Second, challenges in establishing causality remain unresolved. The gut microbiota-metabolite-host axis involves bidirectional and multi-layered interactions, including microbial community dynamics, host genetics, dietary exposure, and organ-specific metabolism. These interconnected feedback loops complicate traditional causal modeling. Future studies will require longitudinal cohort designs, Mendelian randomization approaches, and well-controlled interventional trials to disentangle causality from correlation.
Third, substantial interindividual variability exists in microbial metabolite production. Differences in gut microbial composition, dietary patterns, age, sex, renal function, medication use (e.g., antibiotics, statins, beta-blockers), and geographic background all significantly influence metabolite levels such as TMAO, SCFAs, and PAGln. This variability limits the generalizability of findings and highlights the need for patient-stratified analyses rather than population-level averages. In parallel, microbiome-informed precision medicine has gained increasing attention in recent literature, focusing on individualized risk prediction and tailored therapeutic interventions based on gut microbial metabolic profiles (118).
Fourth, standardization of microbiome and metabolomic methodologies remains a critical unmet need. Current studies employ heterogeneous protocols for sample collection, storage, sequencing platforms, metabolite quantification, and bioinformatic pipelines. This methodological inconsistency significantly impairs cross-study comparability and reproducibility. Establishing standardized operating procedures and consensus reference ranges for key metabolites is essential for clinical translation.
Fifth, robust multicenter validation studies are urgently required. Most existing evidence derives from single-center or small-scale cohorts with limited ethnic and geographic diversity. Large, prospective, multicenter studies with harmonized endpoints are necessary to validate microbial metabolites as reliable biomarkers and to evaluate the efficacy of microbiota-targeted interventions in real-world cardiovascular populations.
Finally, future cardiovascular medicine is expected to shift toward microbiome-informed precision strategies. Integration of metagenomic, metabolomic, and clinical data may enable patient stratification based on microbial metabolic phenotypes, allowing individualized dietary interventions, targeted microbial modulation, and metabolite-guided therapeutic decision-making. However, such precision approaches remain in the early exploratory stage and require robust predictive models, clinical validation, and regulatory frameworks before implementation.

Collectively, addressing these knowledge gaps will be essential for translating gut microbiota-derived metabolite research into clinically meaningful cardiovascular applications.

## 7 Conclusions

Gut microbiota-derived metabolites have emerged as key functional mediators linking the intestinal microbiome with cardiovascular physiology and disease. Increasing evidence suggests that these metabolites participate in the development and progression of cardiovascular disorders through multiple interconnected mechanisms, including modulation of inflammatory responses, endothelial function, lipid metabolism, thrombosis, and vascular homeostasis.

Among the major metabolites, TMAO, SCFAs, PAGln, bile acids, and tryptophan-derived compounds represent central nodes of the gut–heart axis. However, the current evidence base is heterogeneous, with most mechanistic insights derived from experimental models and the majority of human studies remaining observational in nature. This highlights a persistent gap between mechanistic plausibility and clinically established causality. The cardiovascular effects of microbial metabolites are highly context-dependent and shaped by host metabolic, genetic, and environmental factors, underscoring the need for stratified and individualized interpretation in future clinical applications.

Overall, gut microbiota-derived metabolites provide a unifying framework for understanding cardiovascular disease as a systems-level disorder driven by host–microbiome–environment interactions. While substantial progress has been made in elucidating underlying biological mechanisms, translation into routine clinical application remains limited and requires further validation in well-designed human studies.
